# The immunostimulatory activity of *Epimedium* flavonoids involves toll-like receptor 7/8

**DOI:** 10.3389/fphar.2025.1514284

**Published:** 2025-04-25

**Authors:** Jingyu Wu, Yi Ou, Min Yao, Jiaquan Liu, Hengxing Ran, Zhengrong Wu, Rihui Wu, Lishe Gan, Dongli Li, Jingwei Jin

**Affiliations:** ^1^ School of Pharmacy and Food Engineering, Guangdong Provincial Key Laboratory of Large Animal Models for Biomedicine, Wuyi University, Jiangmen, China; ^2^ International Healthcare Innovation Institute, Jiangmen, China; ^3^ College of Pharmaceutical Sciences, Zhejiang Chinese Medical University, Hangzhou, China

**Keywords:** Epimedium flavonoids, immunostimulatory activity, vaccine adjuvant, TLR7, TLR8

## Abstract

**Background:**

The flavonoids found in *Epimedium* exhibit a wide range of pharmacological activities, with their immunostimulatory effects emerging as a significant area of research in recent years. However, the underlying mechanism of their immunostimulatory activity remains unclear.

**Purpose:**

To investigate the immunostimulatory effects and elucidate the specific mechanisms of *Epimedium* flavonoids both *in vitro* and *in vivo*.

**Methods:**

The immunostimulatory effects and underlying mechanisms of flavonoids from *Epimedium* were evaluated *in vitro* using a variety of techniques, including cell viability assays, flow cytometry, real-time reverse transcription-quantitative polymerase chain reaction (qRT-PCR), enzyme-linked immunosorbent assay (ELISA), molecular docking, plasmid recombination and transformation, recombinant protein expression, surface plasmon resonance (SPR), and NF-κB/SEAP assays. To investigate the immune response in animal experiments, *Epimedium* flavonoids were compared with traditional adjuvants, utilizing biochemical analysis and flow cytometry.

**Results:**

*Epimedium* flavonoids, primarily composed of icaritin, icariin I and icariin II, were observed to significantly enhance the expression of surface co-stimulatory molecules (CD40, CD80, CD86) and major histocompatibility complex (MHC-I, MHC-II) in bone marrow-derived dendritic cells (BMDCs) and RAW 264.7 cells. Additionally, the production of chemokines and pro-inflammatory cytokines was significantly increased in RAW 264.7 cells. *In vivo*, the findings demonstrated that the vaccine adjuvant containing *Epimedium* flavonoids significantly increased the serum concentration of total OVA-specific IgG compared to the control group. SPR analysis revealed that icariin II exhibited the highest binding response to TLR7, while icariin I and icariin II showed the strongest interactions with TLR8 protein, even surpassing the positive control drug, Resiquimod. The NF-κB/SEAP assay further confirmed that icaritin, icariin I, and icariin II enhanced NF-κB activity and stimulated SEAP secretion through TLR7/8 activation.

## 1 Introduction

Vaccination is one of the most effective strategies for preventing and treating infectious diseases (Goff et al., 2015). The primary objective of vaccination is to elicit a robust and targeted immune response that provides long-lasting protection against infection. Additionally, it aims to stimulate the immune system to develop adaptive immunity against pathogens (Verma et al., 2023). Early vaccine formulations were often impure and contained extraneous antigens, which compromised their effectiveness. The advent of recombinant DNA technology and synthetic chemistry has facilitated the production of highly purified antigens (Liu X. et al., 2021), allowing for a more precise and targeted immune response. However, a major drawback of vaccines composed solely of purified antigens is their reduced immunogenicity (Chilamakuri and Agarwal, 2021). As a result, these antigenic formulations often require the inclusion of adjuvants to enhance immunogenicity and elicit a protective immune response (Takahama and Yamamoto, 2020).

Adjuvants are employed to enhance and modulate the immunogenicity of vaccines without directly inducing a specific immune response (Chen H. et al., 2024). Most subunit vaccines rely on adjuvants to enhance their efficacy; however, the development of novel adjuvants has progressed relatively slowly. To date, only a limited number of adjuvants have been approved for human use (Liu T. et al., 2021). Researchers are actively exploring more effective adjuvants with reduced adverse effects, improved ease of synthesis, and lower production costs (Wei et al., 2023). In this context, scientists have recently been investigating the potential of natural adjuvants, including traditional Chinese herbs.

Traditional Chinese herbal remedies contain a diverse array of bioactive compounds, including flavonoids, glycosides, polysaccharides, acids, terpenes, polyphenols, and alkaloids (Zebeaman et al., 2023). Among these, flavonoids represent a significant class of bioactive compounds with a broad spectrum of pharmacological activities, most notably their immunomodulatory effects. In traditional Chinese medicine, flavonoids regulate the immune system by binding to various receptors on immune cells and activating distinct signaling pathways. The host immune system recognizes microorganisms, including viruses, bacteria, and fungi, primarily through the detection of conserved molecular structures known as pathogen-associated molecular patterns (PAMPs).

*Epimedium* is one of the most well-known Chinese medicinal herbs, first documented in the Shenlong Materia Medica between the 2nd and 3rd centuries CE (Wang, 2021). With a long history of clinical application, *Epimedium* has been traditionally used to reinforce kidney function and invigorate Yang in traditional Chinese medicine. However, the complexity of interactions among its various components has made it challenging to determine a precise safe dosage, despite previous evidence supporting its safety. This thesis primarily focuses on the screening and analysis of flavonoids present in *Epimedium*, including icariin, icaritin, icariin I, icariin II, Epimedin A, Epimedin B, Epimedin C, and other related compounds (Zhang et al., 2020).

Pattern recognition receptors (PRRs) are germline-encoded receptors that detect pathogen-associated molecular patterns (PAMPs), serving as key upstream regulators of the immune response. Upon pathogen infection, PRRs activate innate immune signaling pathways and induce immune responses, playing a critical role in the initiation of innate immunity (Kawasaki and Kawai, 2014). Based on their structural and functional characteristics, pattern recognition receptors (PRRs) are classified into six families: Toll-like receptors (TLRs), C-type lectin receptors (CLRs), NOD-like receptors (NLRs), RIG-I-like receptors (RLRs), AIM2-like receptors (ALRs), and other receptors (OLRs) (Howard et al., 2022). This paper focuses on the roles of TLR7 and TLR8, which specifically recognize distinct RNA sequences and are functionally localized within endosomes (Wang et al., 2021). Studies conducted in 2011 demonstrated the pivotal role of TLR7 and TLR8 in initiating both innate and adaptive immune responses. These highly conserved proteins interact with a variety of small molecules and nucleic acids. Activation of TLR7/8 plays a crucial role in protecting the host from invading pathogens while enhancing the overall immune response (Huang et al., 2021). However, sustained TLR7/8 signaling can result in an exaggerated immune response, potentially contributing to chronic inflammation and autoimmune disorders (Singh et al., 2014). Therefore, agonists and antagonists targeting the TLR7/8 pathway represent promising therapeutic candidates for the treatment of immune-related diseases (Sun et al., 2022).

TLR7 and TLR8 play pivotal roles in the acquired immune response, and their structural and functional similarities have attracted considerable attention. Ligand binding to TLR7 and TLR8 triggers the activation of NF-κB signaling, leading to the production of pro-inflammatory cytokines and type I interferons (IFNs). These immune mediators enhance the bactericidal activity of leukocytes and promote the maturation and function of antigen-presenting cells (APCs), thereby orchestrating the acquired immune response. A review of the literature suggests that most immunomodulatory drugs of natural origin are polysaccharides, which are characterized by their large molecular structures. While small-molecule compounds with immunomodulatory activity have garnered increasing attention, their initial recognition by pattern recognition receptors and mechanisms of action remain relatively unexplored (Deng et al., 2014). Most of these small molecules are either synthetic drugs or nanomaterials, with relatively few derived from natural products. Investigating the interactions between small-molecule compounds extracted from *Epimedium* and TLR7/8 could enhance our understanding of small-molecule immunomodulation and provide new insights into the immunoregulatory role of traditional Chinese medicine.

## 2 Materials and methods

### 2.1 Materials and reagents

RAW 264.7 macrophages and 293T cells were purchased from Procell (Wuhan, China). Dulbecco’s Modified Eagle Medium (DMEM), RPMI-1640 culture medium, phosphate-buffered saline (PBS), penicillin-streptomycin solution, and tryspin were obtained from Gibco Life Technologies (Waltham, MA, United States). Fetal bovine serum (FBS) was sourced from Cytiva (Shanghai, China). The tested compounds were purchased from Yuanye Bio-Technology Co., Ltd (Shanghai, China). Fluorophore-conjugated antibodies, including PE-CD40, PE-CD80, PE-CD86, PE-MHC-I, PE-MHC-II and PE-CD11c were acquired from Thermo Fisher Scientific (Waltham, MA, United States) and eBioscience (San Diego, CA, United States), IL-4 and IFN-γ enzyme-linked immunosorbent assay (ELISA) were obtained from Universal Biotech Co., Ltd (Shanghai, China). ELISA kits for IL-6, TNF-α, MIP-1α, and MCP-1 were purchased from NeoBioscience Technology Co., Ltd (Shanghai, China). For molecular assays, StarScript III RT kit and 2×RealStar Fast SYBR qPCR Mix were sourced from GenStar (Beijing, China). And the pNF-κB/SEAP kit was obtained from Novus Biologicals (Colorado, United States). Immunoglobulin antibodies (IgG, IgG2a, IgG2b, IgG1, IgG3 and IgE) were sourced from Abcam (Hong Kong, China).

### 2.2 Cell culture

RAW 264.7 and 293T-cell were cultured in DMEM medium supplemented with 10% FBS, 100 μg/mL streptomycin, and 100 units/mL penicillin at 37°C in a 5% CO_2_ incubator.

### 2.3 Cell viability

RAW 264.7 cells were seeded in 96-well plate at a density of 1 × 10^5^ cells/well and incubated for 24 h. After removing the old medium, 100 μL of *Epimedium* flavonoids at varying concentrations (10, 20, 30, 40 μM) was added to each well, followed by incubation for an additional 24 h. Subsequently, 10 μL of MTT solution (5 mg/mL) was added to each well and incubated for 4 h. 100 μL of DMSO was added to dissolve the formazan crystals and absorbance was measured at 550 nm using a microplate reader.

### 2.4 Flow cytometry

RAW 264.7 cells were seeded in a 12-well plate at a density of 2.5 × 10^5^ cells/well and incubated for 24 h. After removing the old medium, 20 μM *Epimedium* flavonoids were added to each well and incubated for another 24 h. IFN-γ (30 ng/mL) was used as a positive control. The cells were then washed three times with PBS. Subsequently, cells were incubated with 100 μL of 0.05% BSA containing PE-CD80, PE-CD86, PE-CD40, PE-MHC-I, and PE-MHC-II for 30 min. After incubation, the cells were washed 2–3 times with PBS and analyzed by flow cytometry.

### 2.5 The mRNA isolation and qRT-PCR

RAW 264.7 cells were seeded in a 6-well plate at 5 × 10^5^ cells/well and incubated at 37°C for 24 h. The cells were then treated with 20 μM icaritin, icariin I, icariin II for an additional 24 h. LPS (2 μg/mL) was used as positive control. Following Trizol lysis, total RNA was extracted and reverse transcribed into cDNA. qRT-PCR was performed to quantify the expression of inflammatory factors, with all data normalized to the control group. Relative mRNA expression was determined using the 2^−ΔΔCT^ method.

### 2.6 BMDCs and flow cytometry

Bone marrow cells were isolated from the femur of 6-8-week-old C57BL/6 mice and differentiated into BMDCs using GM-CSF (20 ng/mL) and IL-4 (10 ng/mL). Immature BMDCs were harvested on day 7. BMDCs were seeded in a 12-well plate at 1 × 10^6^cells/well and treated with 20 μM icaritin, icariin I, icariin II for 24 h. LPS (2 μg/mL) served as positive control. Cells were then incubated with PE-CD11c, PE-CD40, PE-CD80, PE-CD86, PE-MHC-I and PE-MHC-II at 4 °C in the dark, followed by flow cytometry analysis.

### 2.7 Animal experiments and materials

#### 2.7.1 Animals

BALB/c mice were purchased from Zhuhai BesTest Bio-Tech (Zhuhai, China). Adult mice were housed in a controlled laboratory environment at 23°C ± 1°C with a 12 h light/dark cycle (lights on at 07:00 AM). Mice had *ad libitum* access to food and water.

#### 2.7.2 Animal grouping and administration

A total of 48 female BALB/c mice (6–8 weeks old) were randomly assigned to one of eight experimental groups: normal saline (NS), OVA group, water-in-oil adjuvant, complete Freund’s adjuvant (CFA), aluminum salt adjuvant, icaritin (20 mg/kg), icariin I (20 mg/kg), icariin II (20 mg/kg). After 7 days of acclimation, mice were immunized once, followed by two additional immunizations every 14 days, totaling three immunizations. Body weight was recorded weekly. Prior to each immunization, 200–300 μL of blood was collected from the tail tip, and 42 days after the first immunization, blood was collected via retro-orbital bleeding. The mice were then euthanized, and the heart, liver, spleen, lungs, and kidneys were harvested for further analysis.

#### 2.7.3 Serum antibody titration test

A 100 μL OVA antigen solution (1 mg/mL) was added to the plate and incubated overnight at 4°C. The next day, diluted serum samples were added and incubated at 37°C for 1 h, followed by plate sealing. Subsequently, secondary antibodies (IgG, IgG1, IgG2a, IgG2b, IgG3, IgE) were respectively added and incubated at 37°C for 1 h. TMB substrate solution was then added and incubated in the dark at room temperature for 15–30 min. Finally, absorbance was measured at 450 nm to complete the experiment.

#### 2.7.4 Lymphocyte proliferation and cytokines detection in mouse spleen

The mice were dissected under sterile conditions, the spleens were removed and weighed. The spleens were homogenized and centrifuged, after which the supernatant was discarded. Spleen cells were obtained by resuspending in 1,640 medium, and the cell concentration was adjusted to 5 × 10^6^cells/mL. For the proliferation assay, 100 μL/well of the spleen cell suspension was seeded into 96-well plates, and the experimental groups were stimulated with 5 μg/mL ConA, 10 μg/mL LPS, or 40 μg/mL OVA, respectively. For cytokine analysis, 500 μL of spleen cells was inoculated into 48-well plates, with the experimental group stimulated with 40 μg/mL OVA and the control group treated with PBS. After 48 h of incubation, spleen cell proliferation and cytokine secretion were assessed using CCK-8 and ELISA.

#### 2.7.5 Pathological tissue analysis

The hearts, livers, spleens, lungs, and kidneys of the mice were excised and fixed in 4% paraformaldehyde. Then tissues were paraffin-embedded, sectioned, and stained with hematoxylin-eosin (HE). Subsequently, the stained sections were examined under microscopes at various magnifications and photographed for documentation.

### 2.8 Molecular biology

#### 2.8.1 Computer simulation of molecular docking

Molecular docking experiments were performed using MOE software, where the three molecules icaritin, icariin I, and icariin II were analyzed. TLR7 and TLR8 were selected as the receptor proteins for docking calculations.

#### 2.8.2 Protein expression and purification

Using Snap Gene software, five pairs of primers were designed based on the vector and gene sequence (Supplementary Table S1). The synthesized gene served as a template for amplification. The TLR7 and TLR8 genes were cloned into pMAL-p5x, pMAL-c5x and pGEX-2T vectors via homologous recombination. The recombinant vectors were introduced into *Escherichia coli* to facilitate the expression of the Toll-like receptor 7/8 (TLR7/8) proteins in the supernatant. Subsequently, the TLR7/8 proteins were isolated and purified.

#### 2.8.3 Surface plasmon resonance (SPR)

The affinity of recombinant mouse TLR7 and TLR8 proteins for 80 μM of the small molecules Resiquimod, icaritin, icariin I, and icariin II was assessed using a Biacore S200 system.

### 2.9 NF-κB/SEAP assay

During the logarithmic growth phase, HEK 293T cells were seeded at a density of 1 × 10^5^ cells per well in a 12-well plate. After 24 h, the cells were transfected with pcDNA3.1-TLR7, pcDNA3.1-TLR8 and p-NF-κB/SEAP plasmids. Following an additional 24 h incubation, the cells were treated with icaritin, icariin I and icariin II. After another 24 h, the supernatant was collected, and SEAP secretion was measured to evaluate its activity.

### 2.10 Statistical analysis

Statistical analysis was performed using Prism 10 software (GraphPad, San Diego, CA, United States), and results were expressed as mean ± SD. Differences among experimental groups were assessed using one-way ANOVA, followed by either the least significant difference (LSD) test or Dunnett’s T3 *post hoc* test. Statistical significance was set at p < 0.05.

## 3 Results

### 3.1 Effect of *Epimedium* flavonoids on cell viability and expression of surface co-stimulatory molecules and histocompatibility complexes in RAW 264.7 macrophages

**FIGURE 1:**
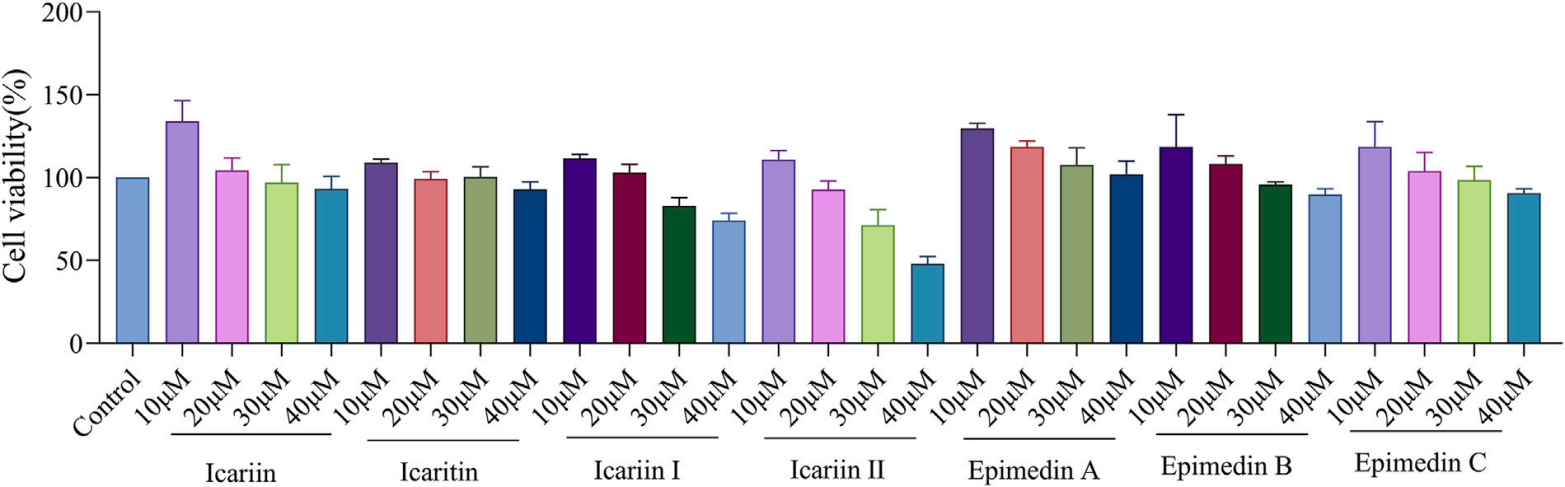
Effect of *Epimedium* flavonoids on cell viability of RAW 264.7 cells. Cell viability was assessed 24 h after compounds treatment in RAW 264.7 cells. The experiment was repeated three times for consistency. Statistical analyses were performed using one-way ANOVA, followed by Tukey’s multiple comparisons test to adjust for multiple comparisons. All data are expressed as mean ± SD (n = 3). Statistical significance is indicated as follows: ****p < 0.0001, ***p < 0.001, **p < 0.01, *p < 0.05, compared with the control group.

The results indicated that none of the seven selected *Epimedium* flavonoids exhibited toxic or adverse effects on RAW 264.7 cells at a concentration of 20 μM. However, at concentrations of 30 μM and 40 μM, icariin I and icariin II induced mild cytotoxic effects on RAW 264.7 cells. Notably, icariin II caused nearly 50% cell death at 40 μM (Figure 1). To ensure the safety and consistency of subsequent experiments, the concentration of *Epimedium* flavonoids was uniformly set to 20 μM for all cell assays.

Macrophages are unique in that they function as both innate immune cells and professional antigen-presenting cells (APCs). Upon stimulation, macrophages upregulate the expression of antigen-presenting proteins on their cell surface, including major histocompatibility complex (MHC) molecules (MHC-I and MHC-II) and co-stimulatory molecules (CD40, CD80 and CD86).

**FIGURE 2:**
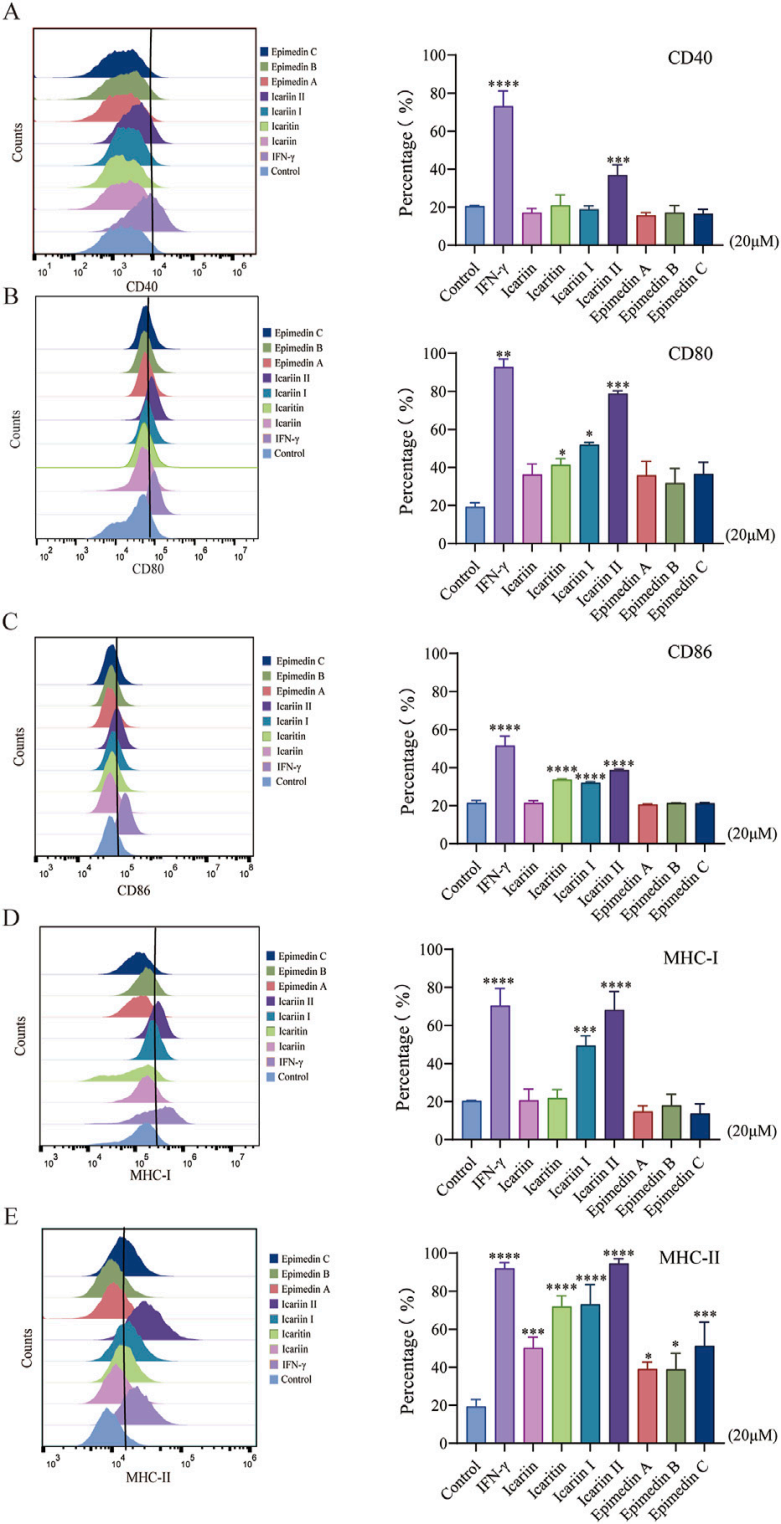
Effects of *Epimedium* flavonoids on the maturation of RAW 264.7 cells. Cell was assessed 24 h after compounds treatment in RAW 264.7 cells. The experiment was repeated three times for consistency. **(A–C)**. Effects of *Epimedium* flavonoids on the expression of CD40, CD80, and CD86, and **(D, E)** effects on MHC-I and MHC-II in RAW 264.7 cells, as detected by flow cytometry. RAW264.7 cells were gated on size and complexity using a two-dimensional FSC vs. SSC plot, then assessed for MHC-I, MHC-II, CD40, CD80 and CD86. Statistical analyses were performed using one-way ANOVA, followed by Tukey’s multiple comparisons test to adjust for multiple comparisons. All data are expressed as mean ± SD (n = 3). Statistical significance is indicated as follows: ****p < 0.0001, ***p < 0.001, **p < 0.01, *p < 0.05, compared with the control group.

Flow cytometry was used to evaluate the expression levels of co-stimulatory molecules and major histocompatibility complex (MHC) molecules on the surface of RAW 264.7 cells following drug treatment. As depicted in Figure 2, *Epimedium* flavonoids enhanced the expression of co-stimulatory molecules and MHC molecules. Specifically, icaritin, icariin I and icariin II significantly increased the expression levels of MHC-I, MHC-II, CD40, CD80 and CD86. Notably, icariin II induced MHC-I and MHC-II expression was comparable to that of the positive control, LPS. The effect of icariin I was less pronounced than expected, possibly due to its larger molecular weight, which may affect its cellular absorption. Based on these findings, icaritin, icariin I and icariin II were selected for further investigation.

### 3.2 The expression of costimulatory molecules in BMDCs induced by icaritin, icariin I and icariin II

**FIGURE 3:**
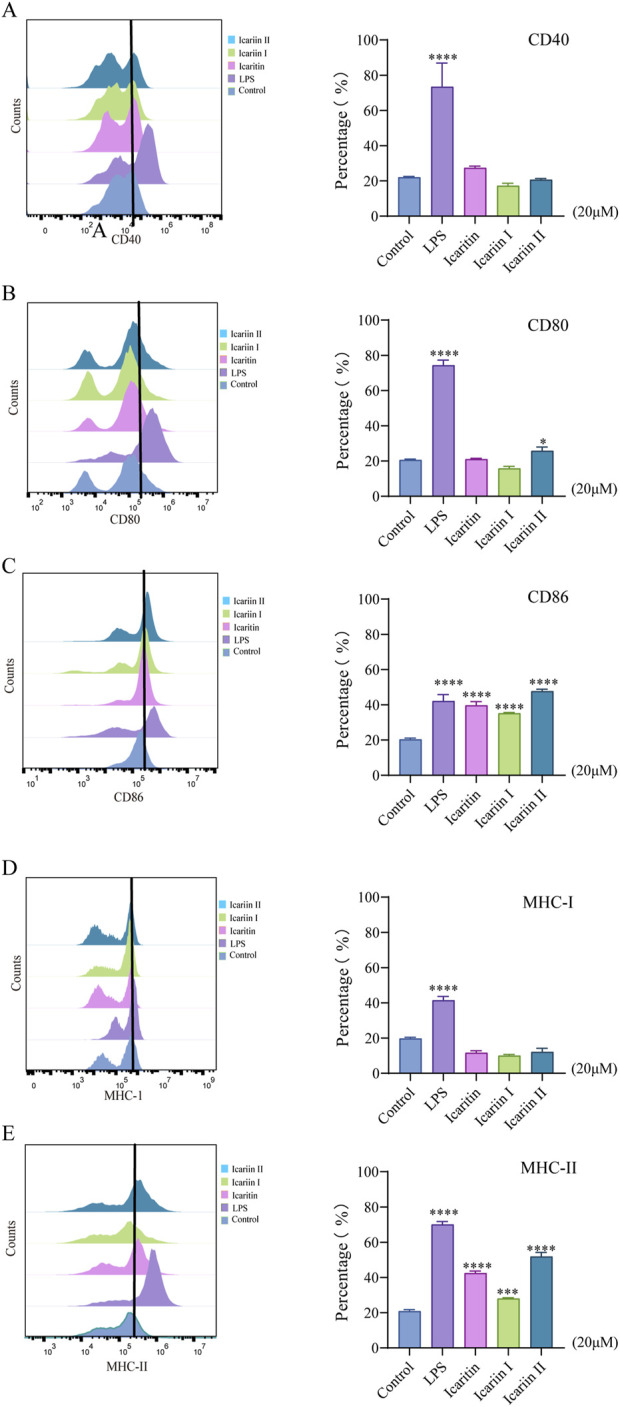
Effects of icaritin, icariin I and icariin II on the maturation of BMDCs. Cell was assessed 24 h after compounds treatment in BMDCs. The experiment was repeated three times for consistency. **(A–C)** Effects of *Epimedium* flavonoids on the expression of CD40, CD80, and CD86, and **(D, E)** effects on MHC-I and MHC-II in BMDCs, as assessed by flow cytometry. BMDCs cells were selected using a two-dimensional FSC vs. SSC plot, followed by gating on CD11c for DCs. Then assessed for MHC-I, MHC-II, CD40, CD80 and CD86. Statistical analyses were performed using one-way ANOVA, followed by Tukey’s multiple comparisons test to adjust for multiple comparisons. All data are presented as mean ± SD (n = 3). Statistical significance is indicated as follows: ****p < 0.0001, ***p < 0.001, **p < 0.01, *p < 0.05, compared with the control group.

Antigen-presenting capability is a key metric for evaluating the efficacy of adjuvants. CD11c serves as a molecular marker for dendritic cells (DCs), while MHC-I, MHC-II, CD80, CD86 and CD40 are crucial indicators of antigen presentation in DCs. The results demonstrated that icaritin, icariin I and icariin II enhanced the expression of CD86 and MHC-II in mouse BMDCs. However, they did not significantly increase the expression of CD40, CD80 and MHC-I (Figure 3). This may be attributed to the role of CD80, which is associated with the persistence and expansion of later T cell responses (Damoiseaux et al., 1998), whereas CD86 plays a critical role in the early T cells co-stimulation, often determining T cell activation (Leifeld et al., 1999; Van Gool et al., 1996). Notably, icariin II elevated CD86 expression to a level even exceeding that of the positive control, LPS. These findings suggest that *Epimedium* flavonoids can promote the maturation and activation of BMDCs. In summary, icaritin, icariin I, and icariin II promote the maturation and activation of various cell lines, enhance antigen-presenting cell activation, and exhibit immunomodulatory effects. Further studies are needed to elucidate the specific immunological mechanisms and explore their potential role in activating antigen-presenting cells (APCs) through the TLR7/8 pathway.

### 3.3 The expression of proinflammatory cytokines and chemokines mRNA levels in RAW 264.7 cells induced by icaritin, icariin I and icariin II

**FIGURE 4:**
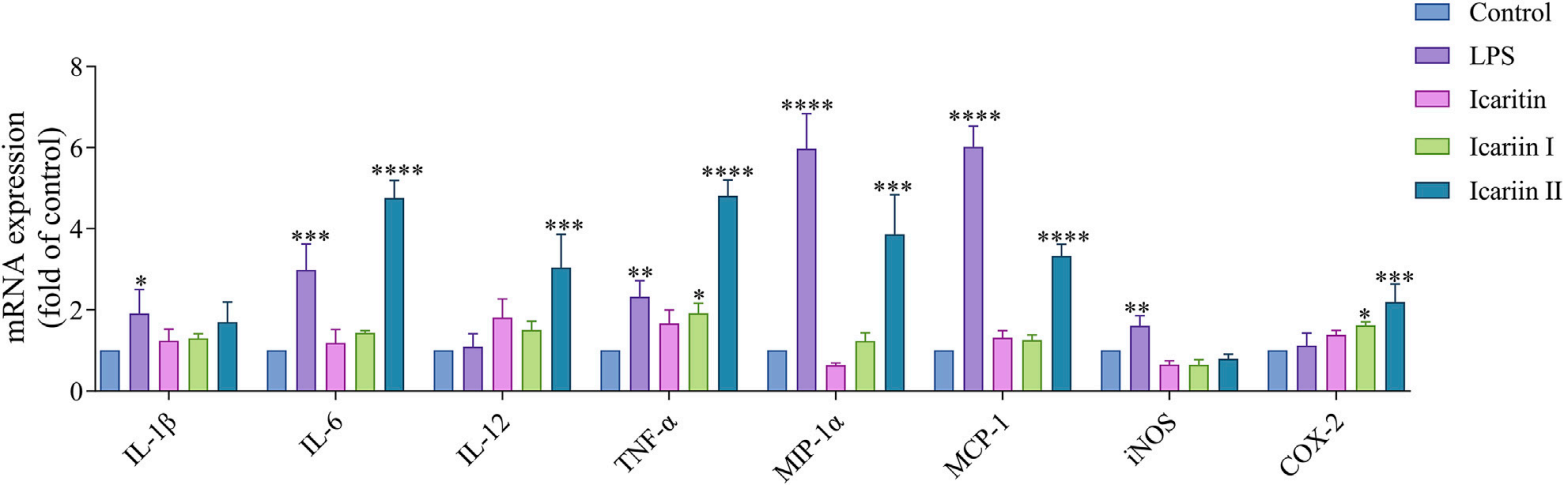
Icaritin, icariin I, icariin II induces an inflammatory response in RAW 264.7 cells. Cell was assessed 24 h after compounds treatment in RAW 264.7 cells. The experiment was repeated three times for consistency. The mRNA levels of IL-1β, IL-6, IL-12, TNF-α, MIP-1α, MCP-1, iNOS and COX-2 in RAW 264.7 cells were examined by qRT-PCR. Statistical analyses were performed using one-way ANOVA, followed by Tukey’s multiple comparisons test to adjust for multiple comparisons. All data are presented as mean ± SD (n = 3). Statistical significance is indicated as follows: ****p < 0.0001, ***p < 0.001, **p < 0.01, *p < 0.05, compared with the control group.

Cytokines and chemokines are essential for immune system function, playing a crucial role in the recruitment and activation of immune cells. As shown in Figure 4, icariin II significantly upregulated the mRNA levels of IL-6, IL-12, TNF-α, MIP-1α, MCP-1 and COX-2 in RAW 264.7 cells. Icariin I increased the mRNA levels of TNF-α and COX-2 in RAW 264.7 cells. In contrast, icaritin did not enhance the mRNA expression of pro-inflammatory cytokines and chemokines.

### 3.4 The expression levels of proinflammatory cytokines and chemokine proteins in RAW 264.7 cells induced by icaritin, icariin I and icariin II

**FIGURE 5:**
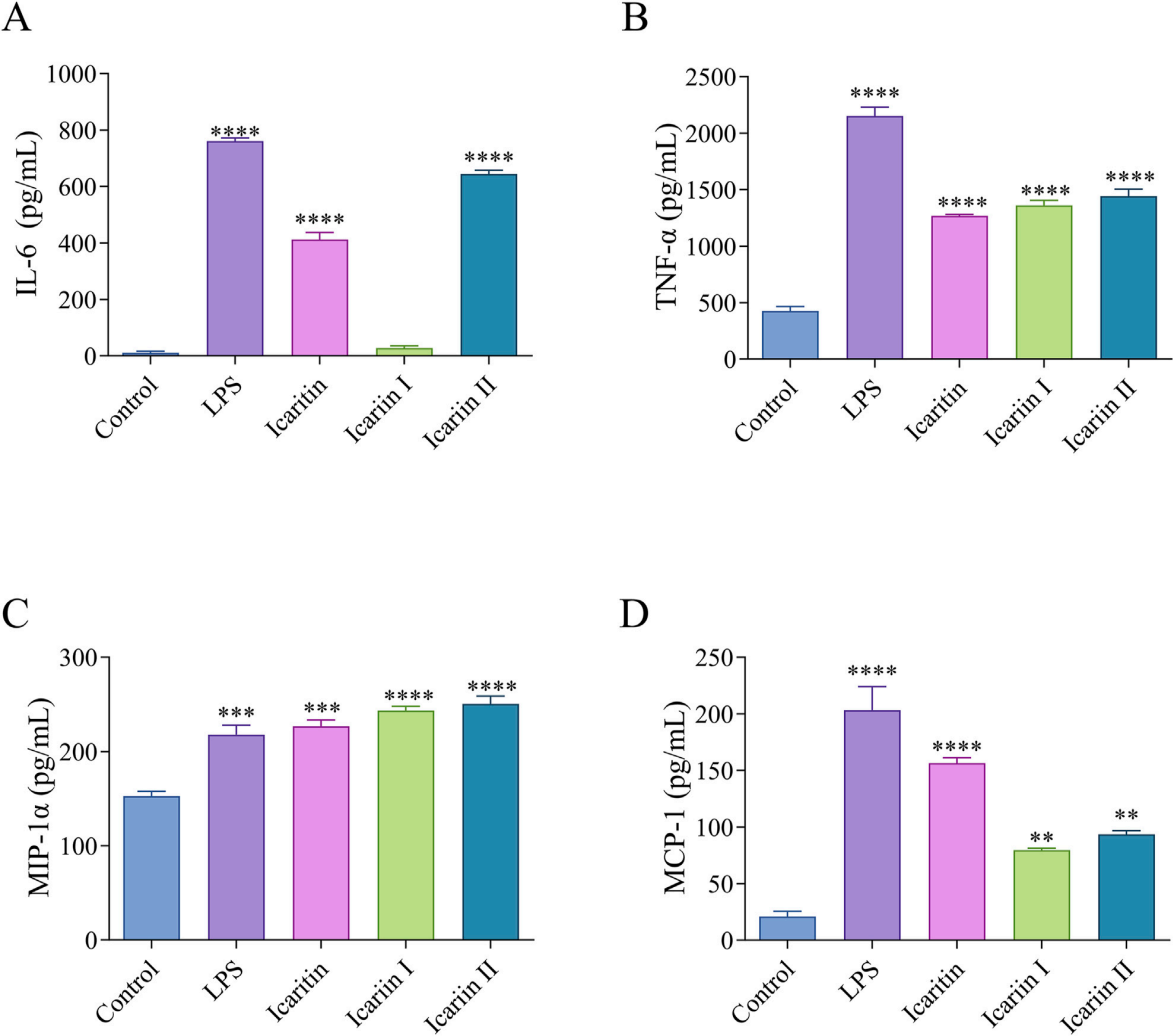
Icaritin, icariin I, icariin II induced an inflammatory response in RAW 264.7 cells. The protein expression levels of **(A)** IL-6, **(B)** TNF-α, **(C)** MIP-1α and **(D)** MCP-1 in RAW 264.7 cells were evaluated by ELISA. Statistical analyses were performed using one-way ANOVA, followed by Tukey’s multiple comparisons test to adjust for multiple comparisons. Values are expressed as mean ± SD (n = 3). Statistical significance is indicated as follows: ****p < 0.0001, ***p < 0.001, **p < 0.01, *p < 0.05, compared with the control group.

Macrophages secrete pro-inflammatory cytokines that enhance T cell responses. To evaluate the effects of *Epimedium* flavonoids on the protein expression of pro-inflammatory cytokines and chemokines in RAW 264.7 cells, the concentrations of these factors in the cell culture supernatant were measured using ELISA kits. As illustrated in Figure 5, icariin Ⅱ significantly increased the secretion levels of IL-6, TNF-α, MIP-1α and MCP-1, which aligns with the qRT-PCR results. Icariin I did not induce a high level of IL-6 protein secretion, a finding consistent with the qRT-PCR results. However, it significantly increased the secretion levels of TNF-α, MIP-1α and MCP-1. Similarly, icaritin also led to a significant increase in the secretion levels of IL-6, TNF-α, MIP-1α and MCP-1. In comparison to the qRT-PCR results, both icaritin and icariin I exhibited discrepancies in secretion patterns, suggesting potential post-transcriptional regulation or translational inefficiencies following treatment with icaritin and icariin I.

### 3.5 The immunoenhancing activity of icaritin, icariin I and icariin II *in vivo*

#### 3.5.1 IgG antibody titer

To evaluate the systemic immune response, the OVA-specific immunoglobulin antibody titer (IgG) antibody titer was measured in mouse serum collected 14 days after each immunization.

**FIGURE 6:**
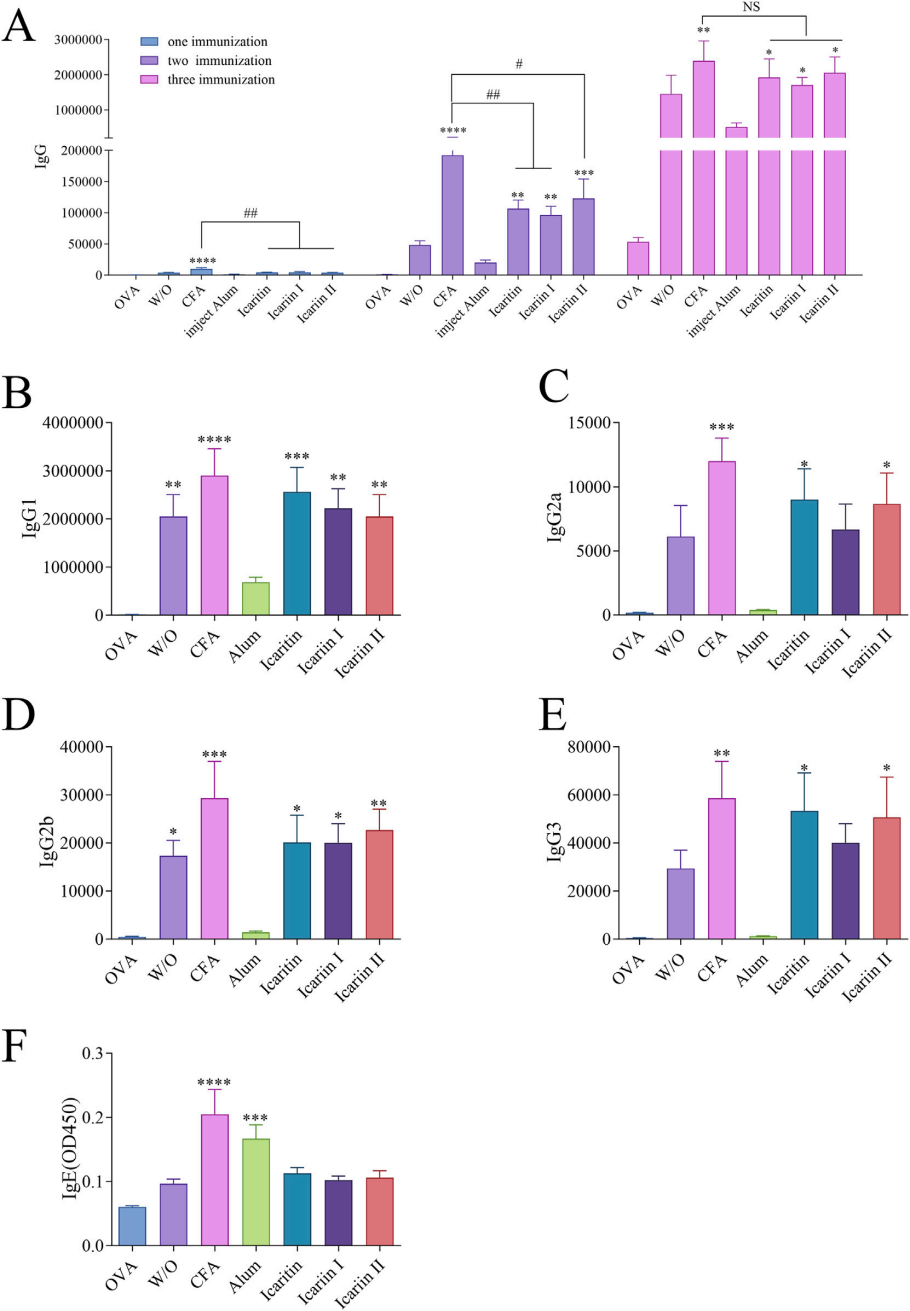
Serum levels of antigen-specific IgG isoforms in mice after immunization. **(A)** Total IgG levels after three immunizations **(B–E)** Serum levels of antigen-specific IgG subtypes (IgG2a, IgG2b, IgG1 and IgG3) after the third immunization, detected by ELISA. **(F)** Serum levels of IgE after three immunizations, measured by ELISA. Statistical analyses were performed using one-way ANOVA, followed by Tukey’s multiple comparisons test to adjust for multiple comparisons. Values are expressed as mean ± SD (n = 6). Normal saline is NS. Statistical significance is indicated as follows: ****p < 0.0001, ***p < 0.001, **p < 0.01, *p < 0.05, compared with the OVA group. ####*p* < 0.0001, ###*p* < 0.001, ##*p* < 0.01, #*p* < 0.05, compared with the CFA group.

As shown in Figure 6A, the water-in-oil emulsifier group exhibited a significantly stronger immune effect than the group receiving direct OVA antigen injection. Although both the CFA group and the *Epimedium* flavonoid oil emulsion adjuvant groups had higher antibody titers than the oil emulsion adjuvant group without the compound, the differences were not statistically, warranting further investigation.

Subsequently, immune response types were partially characterized. As shown in Figures 6B–E, both the CFA group and *Epimedium* flavonoid oil emulsion adjuvant group significantly increased the IgG1, IgG2a, IgG2b and IgG3 antibody titers, indicating that *Epimedium* flavonoids could induce robust Th1 (IgG2a, IgG2b and IgG3) and Th2 (IgG1) immune responses. In contrast, the aluminum salt adjuvant group only promoted IgG1 titers, suggesting an induced Th2 immune response (Howard et al., 2022).

Additionally, antigen-specific IgE antibody levels induced by each vaccine group were examined, as shown in Figure 6F. With the exception of the CFA and aluminum adjuvant groups, no increase in IgE antibody levels was observed in the other groups. Since IgE is associated with allergic reactions, these findings suggest that icaritin, icariin I, and icariin II are unlikely to trigger allergic responses.

#### 3.5.2 Proliferation of mouse splenic lymphocytes and cytokine secretion levels

**FIGURE 7:**
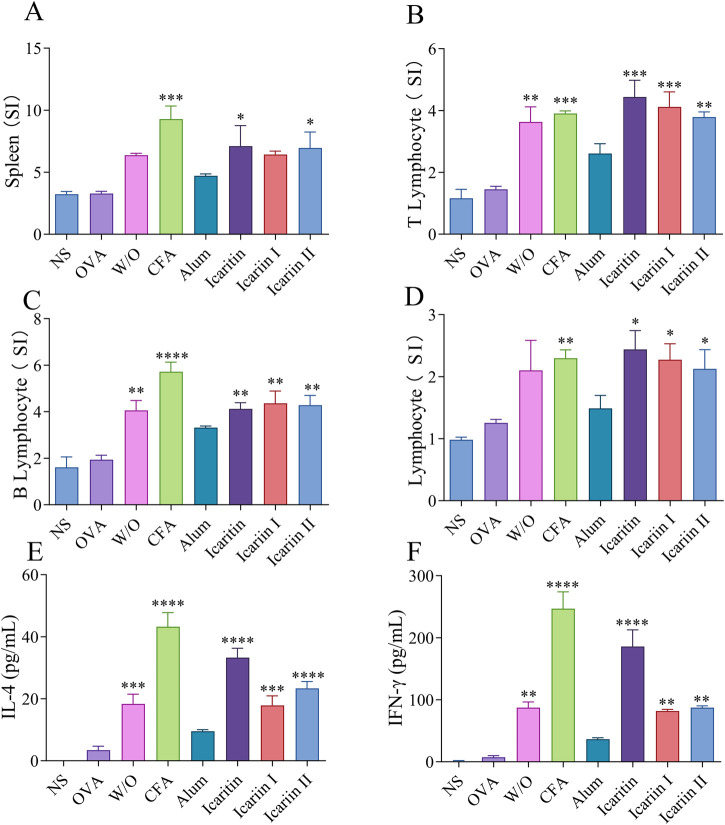
Analysis of splenocytes after the third immunization. **(A)** Spleen organ index **(B)** T-lymphocyte **(C)** B-lymphocyte and **(D)** lymphocyte. Secretion levels of **(E)** IFN-γ and **(F)** IL-4 were detected by ELISA. Statistical analyses were performed using one-way ANOVA, followed by Tukey’s multiple comparisons test to adjust for multiple comparisons. Values are expressed as mean ± SD (n = 3). Statistical significance is indicated as follows: ****p < 0.0001 ***p < 0.001 **p < 0.01 *p < 0.05, compared with the OVA group.

Different cytokine types serve as indicators of Th1 or Th2 biased immune responses. The immune function of T cells was evaluated by analyzing the expression of OVA-specific cytokines in splenic cells. As shown in Figure 7A, the stimulation indices of T lymphocytes, B lymphocytes and total lymphocytes in the icaritin, icariin I and icariin II vaccine group were significantly higher than those in the saline and adjuvant-free groups. Furthermore, the icaritin, icariin I and icariin II vaccine groups significantly enhanced the secretion levels of both IFN-γ and IL-4, with IFN-γ and IL-4 secretion levels in the icariin group comparable to those in the CFA group (Figure 7B). The cytokine secretion results were consistent with the IgG antibody subtype analysis, further indicating that icaritin, icariin I, and icariin II can induce robust Th1 and Th2 immune responses (Shen et al., 2022).

#### 3.5.3 Pathological tissue analysis

**FIGURE 8:**
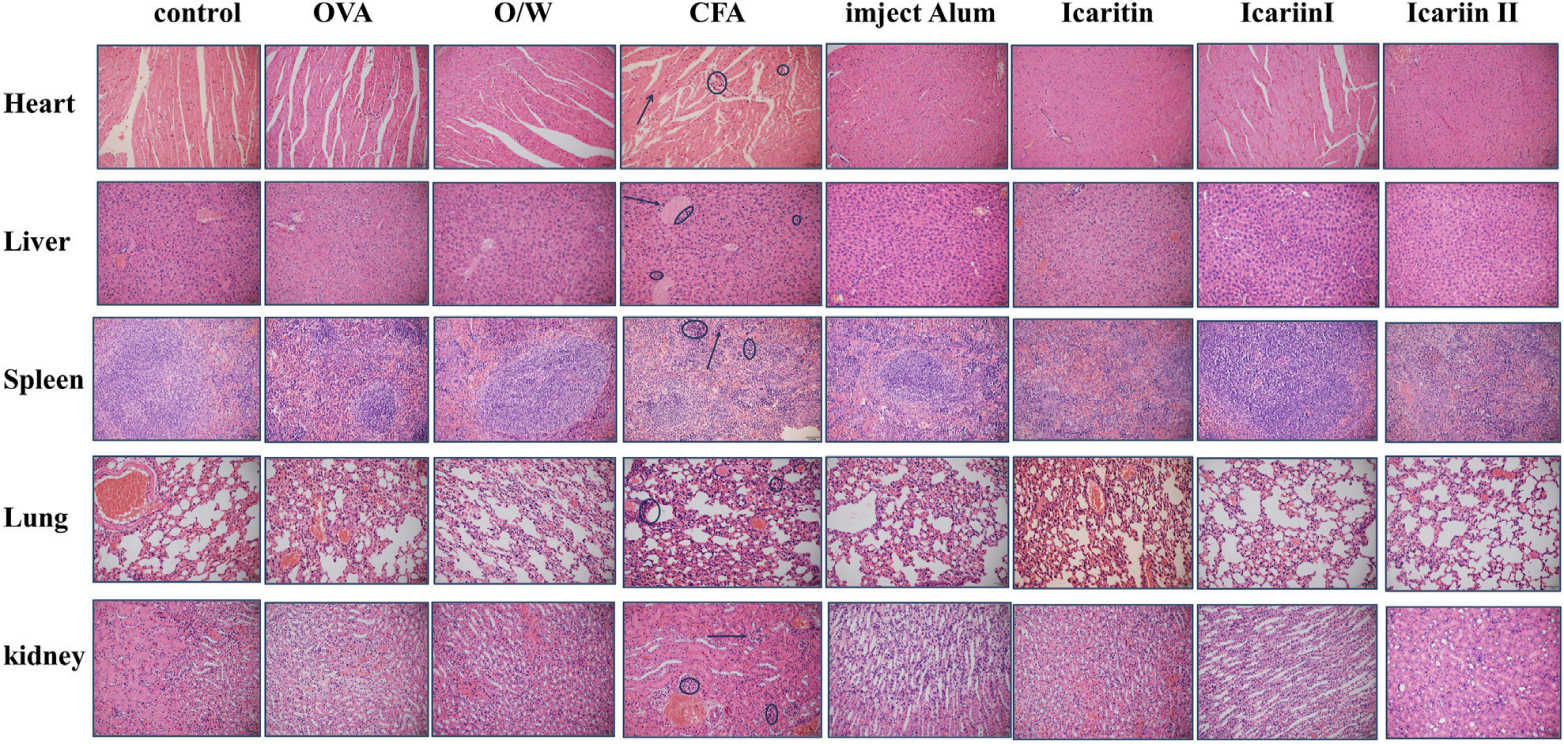
HE staining of major organs of mice after the third immunization. Scale bar = 1 mm, magnification = ×20. Arrows and circles in the figure indicate tissue damage caused by inflammatory cell infiltration (n = 4).

During the experiment, mice in the CFA group exhibited significant swelling at the injection site after immunization, whereas no such swelling was observed in the other groups. To evaluate *in vivo* toxicity, pathological tissue analysis was performed after three immunizations. As shown in Figure 8, inflammatory cell infiltration was observed in the heart, liver, lungs and kidneys of CFA treated mice. In contrast, no significant differences were noted in these organs in the other groups compared to the saline group. These findings suggest that the effective doses of icaritin, icariin I and icariin II did not cause damage to major tissues and organs in mice.

### 3.6 Molecular docking simulation using computers

Computer-simulated molecular docking is a widely used technique for identifying potential targets of compounds and accelerating the screening process for specific molecules. This approach employs flexible and semi-flexible docking methods to evaluate interaction forces between receptors and ligands, thereby predicting the binding modes and affinities of receptor-ligand complexes.

As shown in Supplementary Figures S7–S12, the findings indicate that TLR7 and TLR8 exhibited the strongest interactions with icaritin, icariin I, and icariin II. These interactions were primarily mediated through hydrogen bonding.

### 3.7 The interaction between TLR7 and TLR8 proteins with resiquimod, icaritin, icariin I and icariin II molecules

SPR analysis demonstrated that the pattern recognition receptors (PRRs) targeted by *Epimedium* flavonoids were TLR7 and TLR8. As show in Supplementary Figure S13, icariin I (43 RU) and icariin II (50 RU) exhibited the highest response values with TLR8 protein, with icariin Ⅱ displaying the strongest binding affinity to TLR8 (KD_8_ = 1.03 × 10^−5^ M).

### 3.8 NF-κB/SEAP assay

Secreted placental alkaline phosphatase (SEAP) is a recombinant form of placental alkaline phosphatase used as a reporter for gene function analysis. It is commonly employed to study promoter activity and gene expression in cell cultures and animal sera. NF-κB, a key transcription factor, regulates genes involved in both innate and adaptive immune responses. The activation or inhibition of the NF-κB promoter can be modulated by specific ligands or inhibitors. TLR7/8 ligands activate NF-κB, leading to increased SEAP expression (Caballero et al., 2013; Bender et al., 2020), which is used to evaluate ligand binding to TLR7/8. Resiquimod (R848) exerts immunomodulatory and anti-tumor effects primarily by activating TLR7 and TLR8 on innate immune cells. Therefore, R848 (30 µM) was used as a positive control in this study.

**FIGURE 9:**
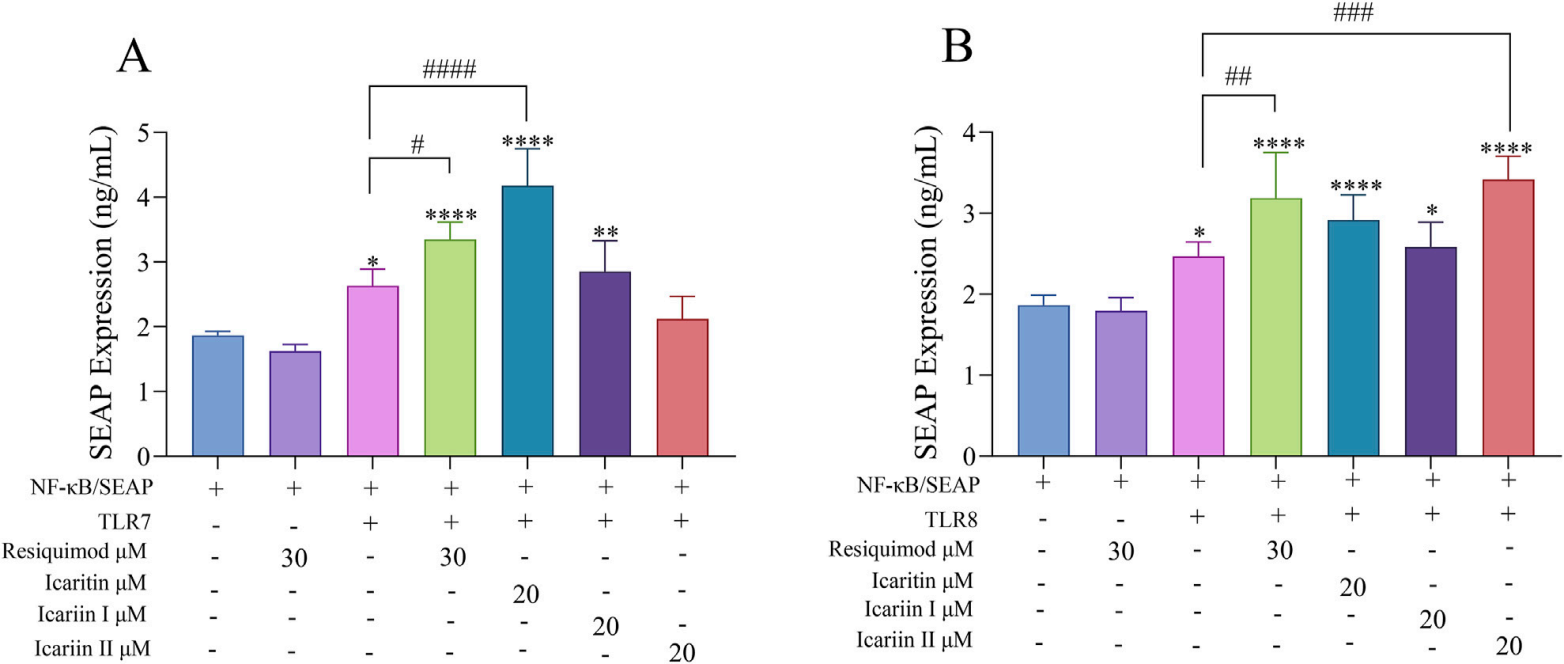
Effects of *Epimedium* flavonoids on SEAP Secretion in Cell supernatants following overexpression of TLR7 and TLR8. Cell was assessed 24 h after compounds treatment in 293T cells. The experiment was repeated three times for consistency. **(A)** TLR7 overexpression. **(B)** TLR8 overexpression. Statistical analyses were performed using one-way ANOVA, followed by Tukey’s multiple comparisons test to adjust for multiple comparisons. Values are expressed as mean ± SD (n = 3). Significant differences are indicated as follows: ****p < 0.0001, ***p < 0.001, **p < 0.01, *p < 0.05, compared with the NF-κB/SEAP group; ####*p* < 0.0001, ###*p* < 0.001, ##*p* < 0.01, #*p* < 0.05, compared with the NF-κB/SEAP + TLR7 group or NF-κB/SEAP + TLR8 group.

As shown in Figure 9A, compared with the NF-κB/SEAP group, the NF-κB/SEAP + TLR7 group, Resiquimod group, icaritin group and icariin Ⅰ group all showed significant differences, suggesting that the TLR7 and NF-κB/SEAP plasmids were successfully transfected and can be stably expressed. However, compared with the NF-κB/SEAP + TLR7 group, only the icaritin group exhibited a significant difference, while the icariin Ⅰ group and icariin Ⅱ did not. This indicates that icaritin can stimulate SEAP secretion by activating the signaling pathway mediated by TLR7, effectively enhancing the activity of NF-κB.

Furthermore, as shown in Figure 9B, the experimental results also demonstrated that, following the overexpression of TLR8 and NF-κB/SEAP, icariin II can remarkably enhance the expression of SEAP. This indicates that icariin Ⅱ can stimulate SEAP secretion by activating the signaling pathway mediated by TLR8 and effectively enhancing the activity of NF-κB.

In conclusion, icaritin was able to specifically interact with TLR7, while icariin II specifically interacted with TLR8. Both compounds activated the downstream NF-κB signaling pathway (Piras and Selvarajoo, 2014), consequently promoting the secretion of SEAP. Therefore, icaritin, icariin I, and icariin II can activate the TLR7/8-NF-κB pathway. Combined with the molecular docking and SPR results discussed earlier, it can be concluded that icaritin, icariin I, and icariin II likely bind to TLR7/8 receptors, leading to the activation of APCs.

## 4 Discussion

Vaccines are among the most effective measures for preventing infectious diseases, and vaccine adjuvants play a crucial role in enhancing their efficacy. Adjuvants amplify the immune response against specific antigens present in vaccines (Pulendran et al., 2021). RAW 264.7 and DCs are common APCs responsible for initiating and regulating both innate and adaptive immune responses. Upon detecting PAMPs or other danger signals, immature RAW 264.7 and DCs upregulate the expression of co-stimulatory molecules and adhesion molecules such as MHC-I, MHC-II, CD40, CD80 and CD86, facilitating their maturation. Fully mature DCs also secrete various cytokines, including IL-6 and TNF-α, further modulating the immune response.

*In vitro* activity studies revealed that *Epimedium* flavonoids significantly enhanced the expression of co-stimulatory molecules, MHC-I and MHC-II, while also promoting the expression and secretion of pro-inflammatory cytokines and chemokines, including IL-6, TNF-α, MIP-1α and MCP-1. *In vivo* studies demonstrated that mice immunized with *Epimedium* flavonoids exhibited high levels of total IgG antibodies in their serum, with antibody levels increasing over time and significantly elevating IgG1, IgG2a, IgG2b and IgG3 antibody titers. These findings indicate that *Epimedium* flavonoids can induce robust Th1 (IgG2a, IgG2b and IgG3) and Th2 (IgG1) immune responses. Splenocyte experiments futher confirmed the production of Th1- and Th2-related cytokines, with IFN-γ levels being significantly higher than IL-4, indicating a stronger Th1-biased response. Additionally, recombinant plasmids encoding TLR7 and TLR8 were constructed and overexpressed in 293T cells. NF-κB/SEAP asssays confirmed that *Epimedium* flavonoids exert their immunomodulatory effects through the TLR7/8 pathway. Finally, this study successfully expressed mouse TLR7 and TLR8 proteins using prokaryotic expression systems and confirmed via SPR technology that the PRRs targeted by *Epimedium* flavonoids are indeed TLR7 and TLR8.

In conclusion, icaritin, icariin I and icariin II can recognize receptors through TLR7/8 pattern and exert immunomodulatory effects both *in vivo* and *in vitro* models. Furthermore, they do not cause tissue damage at safe concentrations.

Icaritin, icariin I and icariin II were compared with TLR7/8 agonists imiquimod, resiquimod. Although they are among the most extensively studied TLR7/8 agonists, they exhibit certain limitations. For instance, imiquimod specifically targets TLR7 but is largely ineffective in murine models, and its immunostimulatory capacity is relatively weak. Resiquimod, while capable of activating both TLR7 and TLR8 in humans, lacks activity toward murine TLR8, making it challenging to accurately model its effects in mice. Moreover, due to its high potency, resiquimod may induce cytotoxicity and even trigger excessive immune responses such as cytokine storms (Dockrell and Kinghorn, 2001).

In contrast, icaritin, icariin I, and icariin II, as natural compounds derived from traditional Chinese medicine, exhibit favorable biosafety profiles and broader therapeutic windows (Yong et al., 2021). When administered subcutaneously in mice, these compounds do not induce noticeable local inflammatory responses such as redness, swelling, or pain, indicating good tolerability. More importantly, they demonstrate a strong affinity for both TLR7 and TLR8, effectively activating immune signaling pathways while eliciting relatively mild immune reactions. These characteristics suggest promising potential for their application as novel, safe, and effective immunomodulatory agents.

These findings provide theoretical support for the development of *Epimedium* as an immunomodulatory agent and offer insights into vaccine adjuvant research. Additionally, this study contributes to futher investigations into *Epimedium*, a prominent traditional Chinese herbal medicine. In recent years, cancer immunotherapy has become a hot research topic, particularly in the development of TLR7/8 immunomodulators. The *Epimedium* flavonoid compounds mentioned in this study—icaritin, icariin I, and icariin II—have drawn our attention due to their potential role as TLR7/8 modulators and their anticancer activity. *Epimedium* flavonoid compounds exhibit diverse biological activities, including direct anticancer effects, such as inhibiting tumor proliferation, promoting apoptosis, and suppressing tumor cell invasion and metastasis (Chen Y. et al., 2024; Ding et al., 2024). Additionally, these compounds can modulate the tumor microenvironment (TME) by reducing M2 polarization of tumor-associated macrophages (TAMs), increasing CD8^+^ T cell infiltration, and enhancing antitumor immune responses.

TLR7/8 immunomodulators activate dendritic cells (DCs), promoting antigen presentation and enhancing T cell responses, particularly by increasing CD8^+^ T cell activity (Zhou et al., 2022). They exert antitumor effects via IFN-γ and IL-12-mediated mechanisms, altering the tumor microenvironment (TME), promoting M1 macrophage polarization, and suppressing M2 pro-tumor macrophages (Michaelis et al., 2019). Moreover, when combined with immune checkpoint inhibitors (ICIs), TLR7/8 agonists can improve the immune microenvironment of “cold” tumors (low T cell infiltration), making them more responsive to PD-1/PD-L1 therapies (Smits et al., 2008).

According to our study, icaritin, icariin I, and icariin II are potential TLR7/8 immunomodulators. As natural products, they may offer advantages over conventional TLR7/8 agonists like R848, potentially exhibiting lower toxicity and higher bioavailability (Singh et al., 2014). Therefore, exploring *Epimedium* flavonoids as TLR7/8 immunomodulators for anticancer applications holds great promise—not only for their direct tumor-inhibitory effects but also for their ability to modulate the tumor microenvironment, enhance antigen presentation, and activate systemic antitumor immunity.

## Data Availability

The original contributions presented in the study are included in the article/Supplementary Material, further inquiries can be directed to the corresponding authors.

## Abbreviations

APCs, Antigen presenting cells; CLRs, C-type lectin receptors; CFA, Complete Freund’s Adjuvant; DCs, Dendritic cells; DMSO, Dimethyl sulfoxide; ELISA, Enzyme-linked immunosorbent assay; FBS, Fetal bovine serum; HE, Hematoxylin-eosin; IL, Interleukin; Ig, Immunoglobulins; LPS, Lipopolysaccharide; MCP-1, Monocyte chemotactic protein 1; MHC, Major histocompatibility complex; MIP-1α, Macrophage inflammatory protein 1α; OVA, Ovalbumin; PBS, Phosphate buffered saline; SPR, Surface Plasmon Resonance; Th, T helper cells; TLRs, Toll-like receptors; TNF-α, Tumor necrosis factor α.
