# Supplementary material for: The immunostimulatory activity of Epimedium flavonoids involves toll-like receptor 7/8
Source: Front Pharmacol. 2025 Apr 25;16:1514284. doi: 10.3389/fphar.2025.1514284 (PMC12062745; doi:10.3389/fphar.2025.1514284)
Supplement: Supplementary file 1 [file DataSheet1.docx]

**Supplementary Materials**

**The immunostimulatory activity of *Epimedium flavonoids* involves Toll-like receptor 7/8**

Jingyu Wu^1,2#^, Yi Ou^1,2#^, Min Yao^1,2^, Jiaquan Liu^1,2^, Hengxing Ran^1,2^, Zhengrong Wu^1,2^, Rihui Wu^1,2^, Lishe Gan^1,2,3^, Dongli Li^1,2*^, Jingwei Jin^1,2*^

*1 School of Pharmacy and Food Engineering, Guangdong Provincial Key Laboratory of Large Animal Models for Biomedicine, Wuyi University, Jiangmen 529020, PR China*

*2 International Healthcare Innovation Institute (Jiangmen), Jiangmen 529040, PR China*

*3 College of Pharmaceutical Sciences, Zhejiang Chinese Medical University, Hangzhou 310000, PR China*

*Jingyu Wu, E-mail: 2190564174@qq.com; Yi Ou, E-mail: ouyi1422@163.com*

*# These authors contributed equally to this work.*

* Corresponding authors,Dongli Li, [wyuchemldl@wyu.edu.cn,](mailto:wyuchemldl@wyu.edu.cn,) Jingwei Jin, E-mail: wyuchemjjw@126.com

1. **Materials and reagents**

Trypan Blue dye was purchased from Solarbio (Beijing, China). Thiazole Blue (MTT) was purchased from Sigma-Aldrich (St Louis, MO, USA).Lipopolysaccharide (LPS) was purchased from Sigma (MO, USA).DMSO was purchased in McLin (Shanghai, China). Limulus test kit was purchased from Binendo (Xiamen, China). Ovalbumi (OVA) and complete Freund’s adjuvant (CFA) were purchased from Sigma (MO, USA). Aluminium hydroxide adjuvant was purchased from Thermo (MA, USA). TMB was purchased from McLin (Shanghai, China), concanavin A(CoA) was purchased from Sigma (MO, USA). The MBP affinity chromatography column was purchased from Tiandianhe (Changzhou, China). CM5 chip and amino coupling kit were purchased from GE Healthcare (CA, USA). Plasmid small extraction kit and agarose gel DNA recovery kit were purchased from Tengan (Beijing, China). Detoxi-Gel™ was purchased from Thermo (MA, USA). PET-6×His-TLR7, PET-6×His-TLR8 were purchased from VectorBulider (Guangzhou, China). The vector plasmids of PGEX-27, PMAL-p5x and PMAL-c5x pcDNA3.1-3×FLAG-Puro were purchased from Miaoling Biology (Wuhan, China). The counter plate and cell counter were purchased from Thermo Fisher (MA, USA).

1. **Structural analysis of target proteins**

As illustrated in **Figure S1**, it can be observed that mouse TLR7 and TLR8 are transmembrane proteins, which may influence the transcription and expression of target proteins within the system. Predicting the spatial folding effects of the target proteins revealed that the intracellular and transmembrane regions would not affect the structure and function of these proteins. Consequently, TLR7 (27-837) and TLR8 (24-818) were selected as the primary study fragments，used by IUPred3 . These fragments were expressed and purified. Four vectors, pET28a+, pMAL-p5x, pMAL-c5x, and pGEX-2T, were constructed for recombinant prokaryotic expression in this study.


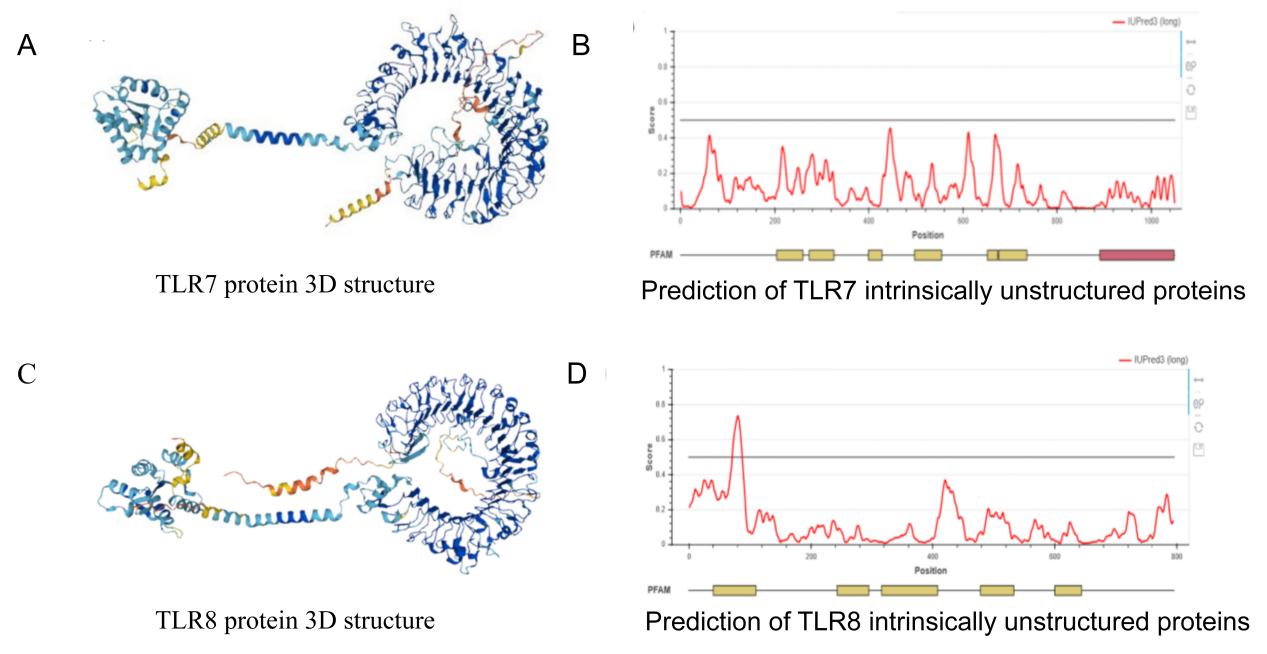
**Figure S1** Structural analysis of target proteins (A) TLR7 protein 3D structure (B) Prediction of TLR7 intrinsically unstructured proteins (C) TLR8 protein 3D structure (D) Prediction of TLR7 intrinsically unstructured proteins

**2. Target Vector Construction**

Using SnapGene software, five pairs of primers were designed according to the vector and gene sequence (**Table S1**). The synthesized gene was used as a template for amplification. The TLR7/8 genes were cloned into pMAL-p5x, pMAL-c5x and pGEX-2T vectors by homologous recombination. The recombinant vector was introduced into Escherichia coli in order to facilitate the expression of the Toll-like receptor 7/8 (TLR7/8) protein supernatant. The subsequent stage of the process involved the isolation and purification of the TLR7/8 protein.

**Table S1** Primer nucleotide sequence

| Primer | Sequence(5’-3’) | Produce (bp) |
| --- | --- | --- |
| p5x(TLR7)-F | gggatcgagggaaggatttcacatgattgcaccgacaagcatctgac | 2367 |
| p5x(TLR7)-R | aattacctgcagggaattcggattaatccagttcgcaggtatacag | 2367 |
| c5x（TLR7)-F | gatcgagggaaggatttcacatgattgcaccgacaagcatctga | 2367 |
| c5x（TLR7)-R | ttatttaattacctgcagggaattaatccagttcgcaggtatac | 2367 |
| pGEX2T(TLR7)-F | ggttccgcgtggatccccgggagattgcaccgacaagcatctga | 2367 |
| pGEX-2T(TLR7)-R | agatcgtcagtcagtcacgatgttaatccagttcgcaggtatac | 2367 |
| c5x（TLR8)-F | agggaaggatttcacataaaaactatagccgcagctatcc | 2388 |
| c5x（TLR8)-R | tttaattacctgcagggaattaggtagtgtcgctcacg | 2388 |

**3. Target protein expression and purification**

After affinity chromatography, the TLR7/8 proteins were subjected to dialysis to remove primary amino components, such as Tris, from the purification process. Ultrafiltration was then used to concentrate the proteins. Following concentration, the proteins were separated and stored at -80°C.

Given the structural and functional similarities between TLR8 and TLR7 proteins, the optimal expression vector, pMAL-c5x, selected for TLR7 expression, was initially employed to construct a recombinant TLR8 protein.

3.1 Plasmid selection

The four constructed vectors were induced to express proteins at 37°C, as illustrated in **Figure S2**. The expression levels of the target proteins from TLR7 pMAL-c5x and pGEX-2T were markedly higher than those from pET28a+ and pMAL-p5x. However, the target proteins formed inclusion bodies, which was disadvantageous for subsequent purification


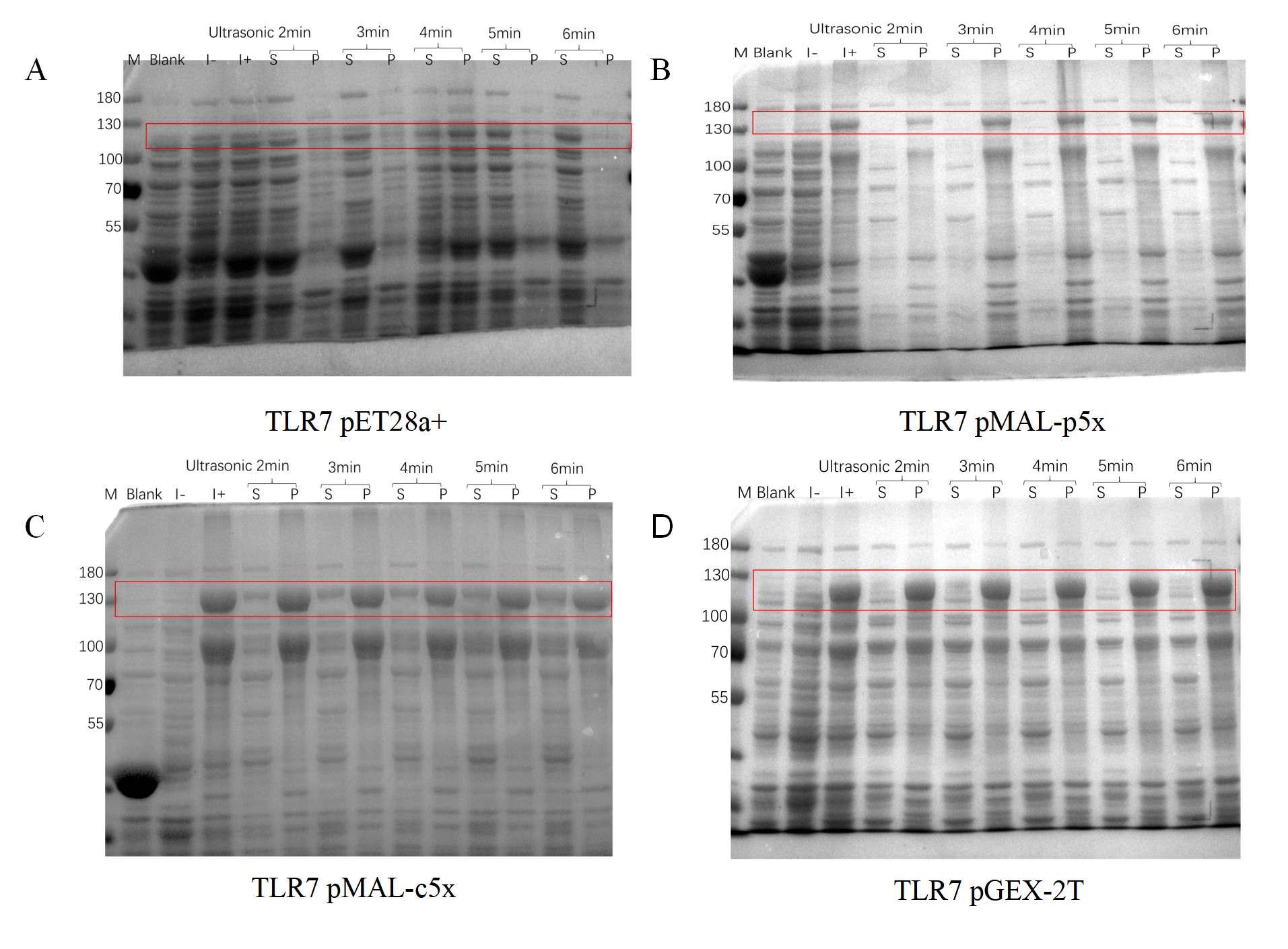


**Figure S2** The expression of carrier proteins.S: supernatant, P: precipitate. (A) TLR7 pET28a+. (B) TLR7 pMAL-p5x. (C) TLR7 pMAL-c5x. (D) TLR7 pGEX-2T.

3.2 OD selection

In the next step, the two vectors with higher expression levels, pMAL-c5x and pGEX-2T, were re-induced to express proteins at 20°C. The expression was evaluated at different initial cell densities (OD values) with IPTG induction, as shown in **Figure S3**. This study found that for TLR7 in the pMAL-c5x vector, the optimal expression of the target protein was achieved when IPTG was added at an OD of 0.7, resulting in the majority of the target protein being secreted into the supernatant.


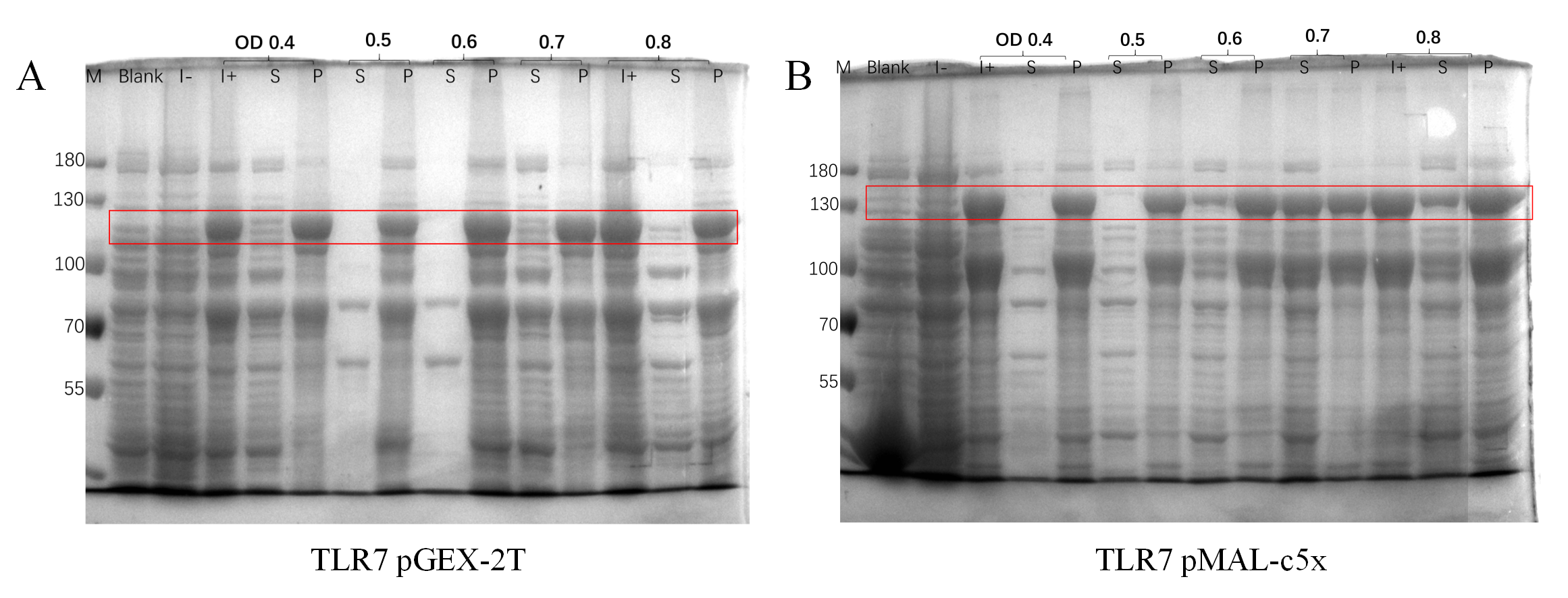


**Figure S3** The expression of carrier proteins.S: supernatant, P: precipitate. (A) TLR7 pGEX-2T (B) TLR7 pMAL-c5x

3.3 Mass preparation of target proteins

As shown in **Figure S4**, TLR7 and TLR8 can be expressed at high levels and secreted into the supernatant.


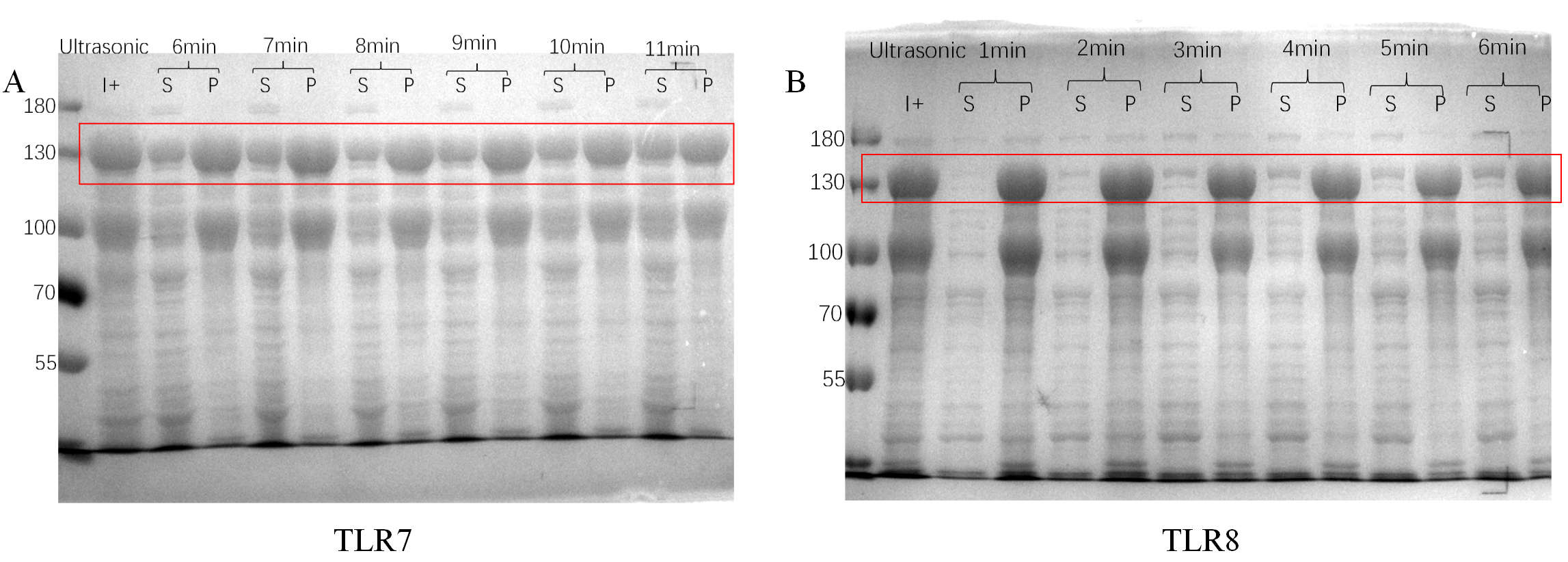


**Figure S4** The expression of carrier proteins.S: supernatant, P: precipitate. The pMAL-c5x vector is produced in large quantities. (A) TLR7 (B) TLR8.

**4. Western blot to confirm target protein**

As demonstrated in **Figure S5**, the protein exhibits a specific binding affinity towards the primary antibody directed against the labeled maltose-binding protein (MBP). This interaction confirms that the protein under investigation is indeed the expressed target protein.


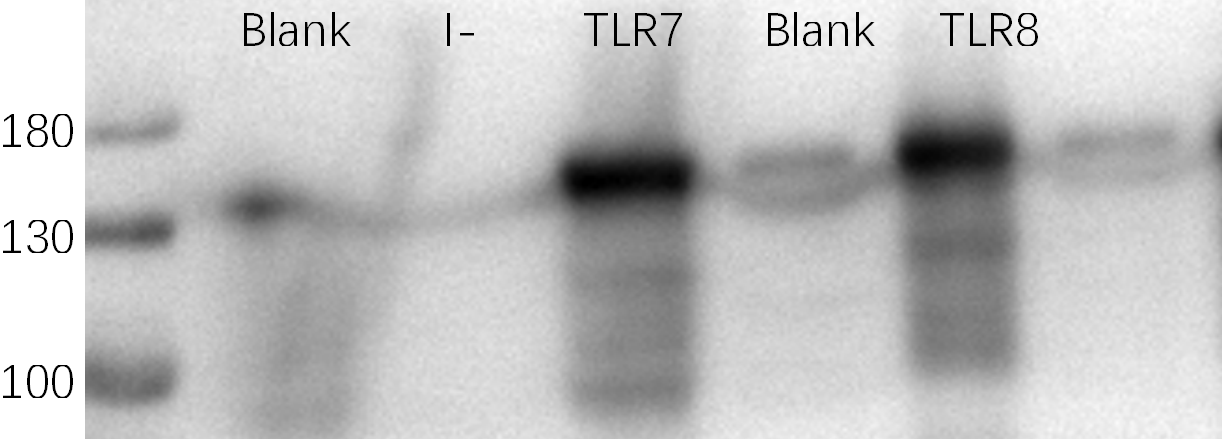


**Figure S5** Western blot analysis was conducted to confirm the presence of the protein.

1. **MBP labeled column affinity chromatography**

Electrophoretic analysis demonstrated that the supernatants containing the pGEX-2T-TLR7 and pGEX-2T-TLR8 proteins were successfully labeled with MBP. Subsequent affinity chromatography results indicated that the target proteins adhered to the column and were eluted efficiently, as illustrated in **Figure S6**.


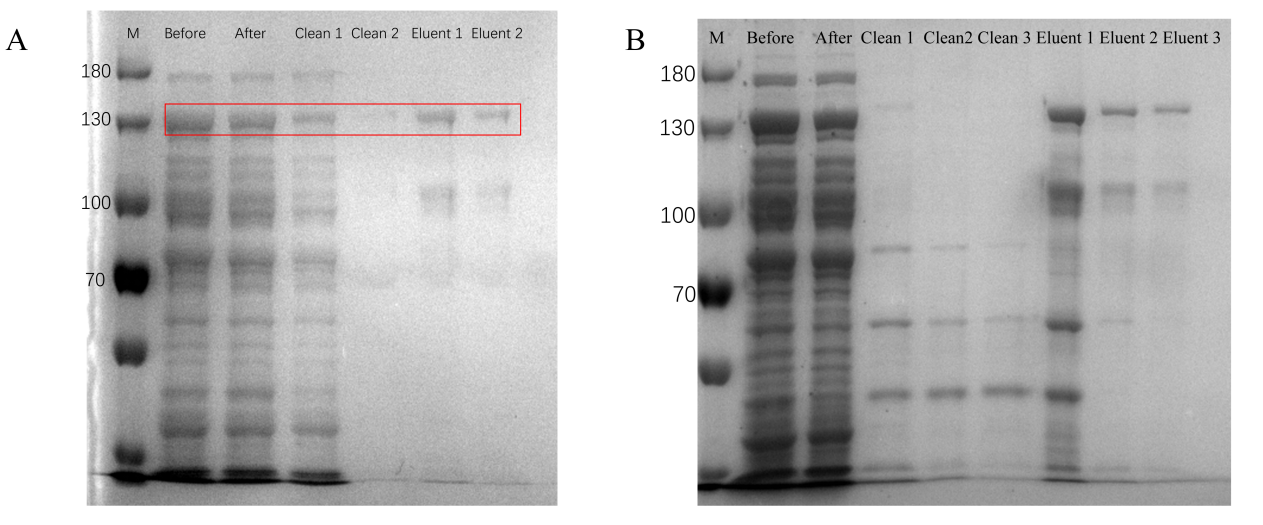


**Figure S6** MBP labeling affinity chromatography column purification, (A) TLR7 (B) TLR8.

**6. Molecular docking simulation using computers**

6.1 The interaction between icaritin and TLR7

The results indicated that the interaction between icaritin and TLR7 primarily relied on four key amino acid residues: arginine (Arg 467), alanine (Ala 472), glutamic acid (Glu 481) and leucine (Leu 105). The scoring value for this interaction was -6.4316 kcal/mol. As shown in **Figure S7**, generally, these amino acid residues played a pivotal role in the interaction, exerting relatively weak dispersive forces with icaritin. This might be the reason for the relatively low scoring value observed.

**
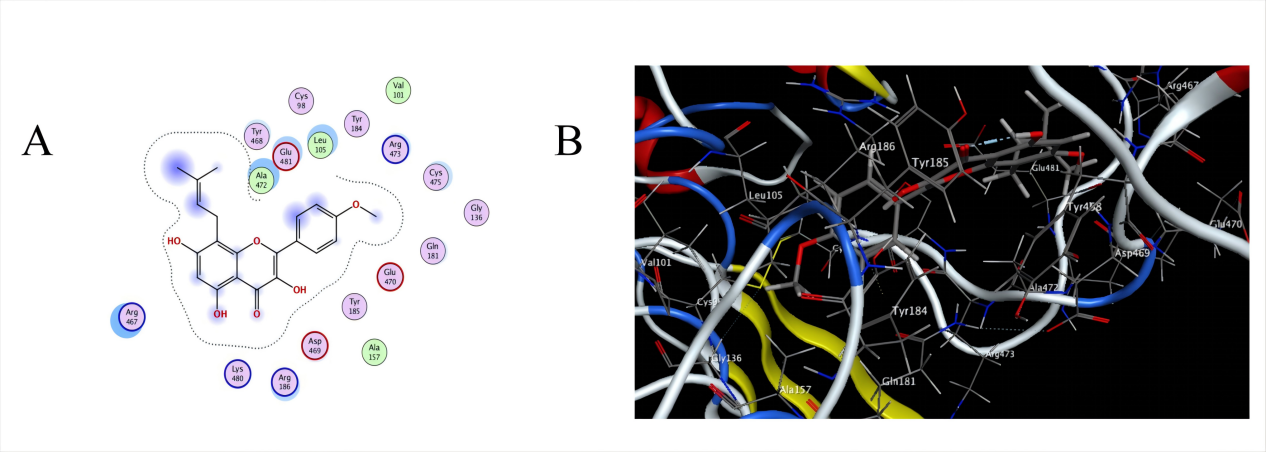
Figure S7** Docking of icaritin to TLR7 receptor protein. (A) 2D conformational map, (B) 3D conformational map

6.2 The interaction between Icariin I and TLR7

The interaction between icariin I and TLR7 was primarily mediated by three key amino acid residues: arginine (Arg 376), aspartic acid (Asp 500), and glutamic acid (Glu 461). With a scoring value of -7.0714 kcal/mol, as shown in **Figure S8**, the side chain of arginine (Arg 376) exhibited a relatively strong electron-donating effect with various hydroxyl groups on the glycosyl moiety. Aspartic acid (Asp 500) displayed an electron-withdrawing effect on the 3-hydroxy group of the flavonoid nucleus. Glutamic acid (Glu 461), on the other hand, interacted with the B-ring of the flavonoid nucleus through weaker dispersive forces.

**
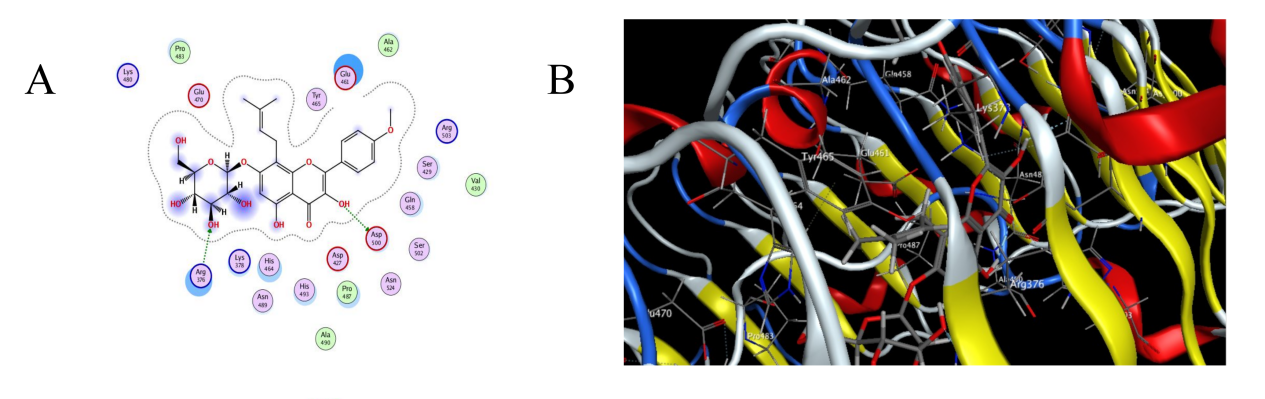
Figure S8** Docking of icariin I to TLR7 receptor protein. (A) 2D conformational map, (B) 3D conformational map

6.3 The interaction between icariin II and TLR7

The interaction between icariin II and TLR7 was primarily mediated by three key amino acid residues: arginine (Arg 628), aspartic acid (Asp 606), and histidine (His 455). With a scoring value of -7.3267 kcal/mol, as depicted in **Figure S9**, the side chain of arginine (Arg 628) exhibited a relatively strong electron-donating effect with various hydroxyl groups on the glycosidic linkage. Aspartic acid (Asp 606) displayed an electron-withdrawing effect on the hydroxyl groups of the glycosyl moiety, creating a synergistic effect that enhances the overall interaction. Additionally, glutamic acid (Glu 461) formed hydrogen bonds with the hydroxyl groups on the A-ring of the flavonoid nucleus.

**
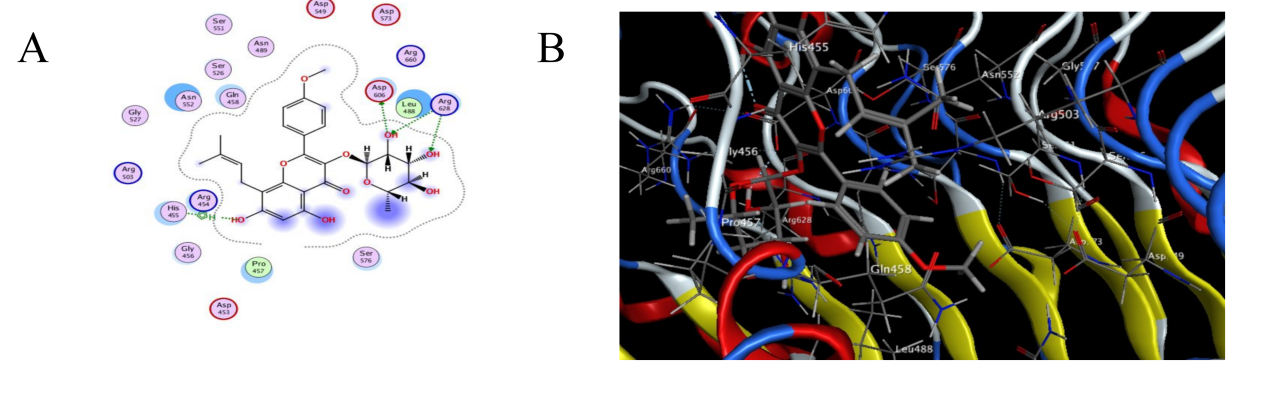
Figure S9** Docking of icariin I to TLR7 receptor protein. (A) 2D conformational map, (B) 3D conformational map

6.4 The interaction between icaritin and TLR8

The interaction between icaritin and TLR8 primarily relied on the key amino acid residues of arginine (Arg 442), lysine (Lys 443), and arginine (Arg 532). The scoring value for this interaction was -6.5959 kcal/mol. As shown in **Figure S10**, generally, these amino acid residues played a pivotal role in the interaction, exerting relatively weak dispersive forces with icariin. Additionally, there were four amino acid residues that exhibited repulsive effects, potentially contributing to the relatively low scoring value observed.

**
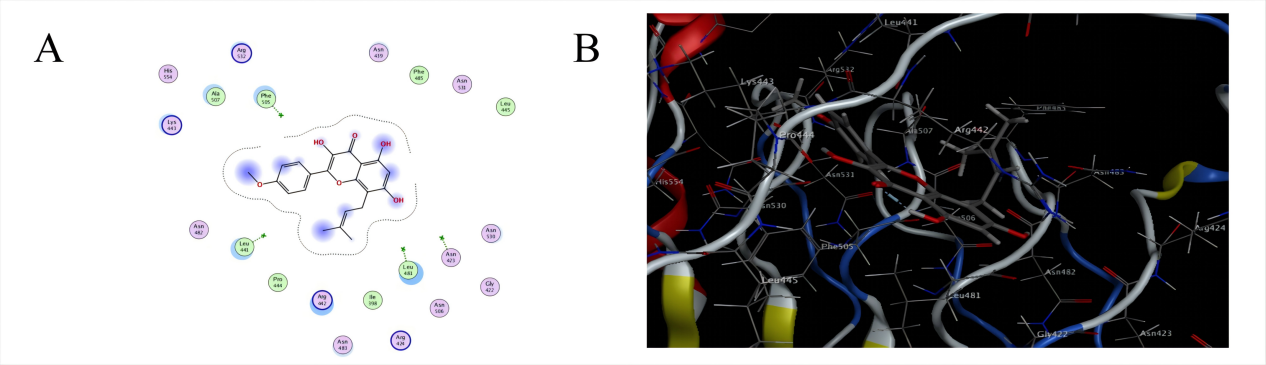
Figure S10** Docking of icariin to TLR7 receptor protein. (A) 2D conformational map, (B) 3D conformational map

6.5 The interaction between icariin I and TLR8

The interaction between icariin I and TLR8 primarily relied on three key amino acid residues: leucine (Leu 481), asparagine (Asn 423), and phenylalanine (Phe 505), with a scoring value of -8.0426 kcal/mol. As shown in **Figure S11**, the interaction between TLR8 and icariin I formed a complete energy ring, indicating a relatively strong and stable interaction. The amino acid residues leucine (Leu 481) and asparagine (Asn 423) on the main chain exhibited electron-withdrawing effects with various hydroxyl groups on the glycosyl moiety. Additionally, phenylalanine (Phe 505) formed hydrogen bonds with the 3-hydroxy group of the flavonoid nucleus. Furthermore, amino acid residues such as lysine (Lys 443), arginine (Arg 532), and arginine (Arg 642) contributed significant dispersive forces to the interaction. The synergistic effect of these various forces created a relatively stable energy system around icariin I, which was likely the reason for the formation of the strongest interaction.


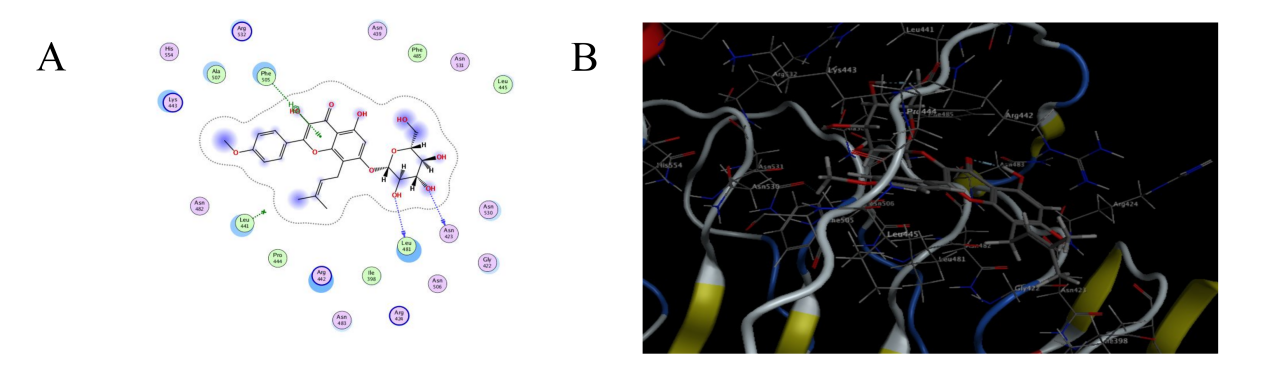


**Figure S11** Docking of icariin II to TLR7 receptor protein. (A) 2D conformational map, (B) 3D conformational map

6.6 The interaction between icariin II and TLR8

The interaction between icariin II and TLR8 primarily relied on the leucine (Leu 441), lysine (Lys 443), and arginine (Arg 442) residues located on the main chain, with a scoring value of -7.6718 kcal/mol. As depicted in **Figure S12**, leucine (Leu 441) exhibited an electron-withdrawing effect with hydroxyl and methyl groups on the glycosyl moiety. Lysine (Lys 443) and arginine (Arg 442) contributed to dispersive forces with icariin II. However, as shown in the **Figure S12**, there were three amino acid residues that exerted repulsive effects, which to some extent weaken the interaction between icariin II and TLR8.


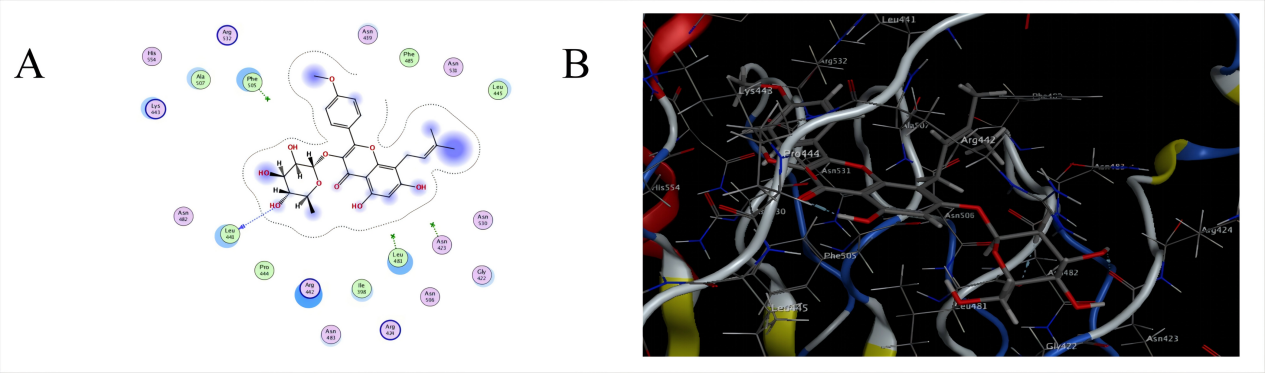


**Figure S12** Docking of icariin II to TLR7 receptor protein. (A) 2D conformational map, (B) 3D conformational map

**7. The interaction between TLR7 and TLR8 proteins with resiquimod, rrcaritin, icariin I and icariin II molecules**

The binding affinities of small molecules, including resiquimod, icaritin, icariin I and icariin II, with TLR7 and TLR8 proteins were measured at different concentrations. A higher response value indicates a better binding specificity between the small molecule and the protein. As shown in **Figure S13**, the recombinant TLR7 and TLR8 proteins were able to specifically bind with the TLR7 and TLR8 agonist resiquimod, with response values reaching 32 RU (KD1 = 5.92×10^-6^ M) and 23 RU (KD5 = 3.67×10^-6^ M), respectively.

Subsequent screenings revealed that icaritin, icariin I and icariin II were all able to specifically bind with TLR7 [with response values of 22 RU (KD2=6.49 × 10^-6^ M), 34 RU (KD3 = 1.61×10^-5^ M), and 45 RU (KD4 = 2.56×10^-5^ M), respectively] and TLR8 [with response values of 42 RU (KD6 = 9.33×10^-8^ M), 43 RU (KD7 = 6.77×10^-6^ M), and 50 RU (KD8 = 1.03×10^-5^ M), respectively]. Notably, icariin I (43 RU) and icariin II (50 RU) exhibited the highest response values with TLR8 protein, even surpassed the response value of the positive control resiquimod (23 RU).

Within the range of 0-80 μM, TLR7 and TLR8 interacted with resiquimod, icaritin, icariin I and icariin II in a concentration-dependent manner. The affinity constant KD, calculated as the ratio of the association constant Ka to the dissociation constant Kd using the Biacore S200 Control Software, provided a measure of the binding strength between the small molecule and the protein. A higher KD value indicates a stronger intermolecular interaction.

According to the affinity constants, icariin II exhibited the strongest interaction with TLR8 (KD8 = 1.03×10^-5^ M), followed by icariin I with TLR8 (KD3 = 1.61×10^-5^ M). These results are generally consistent with the molecular docking experiments.


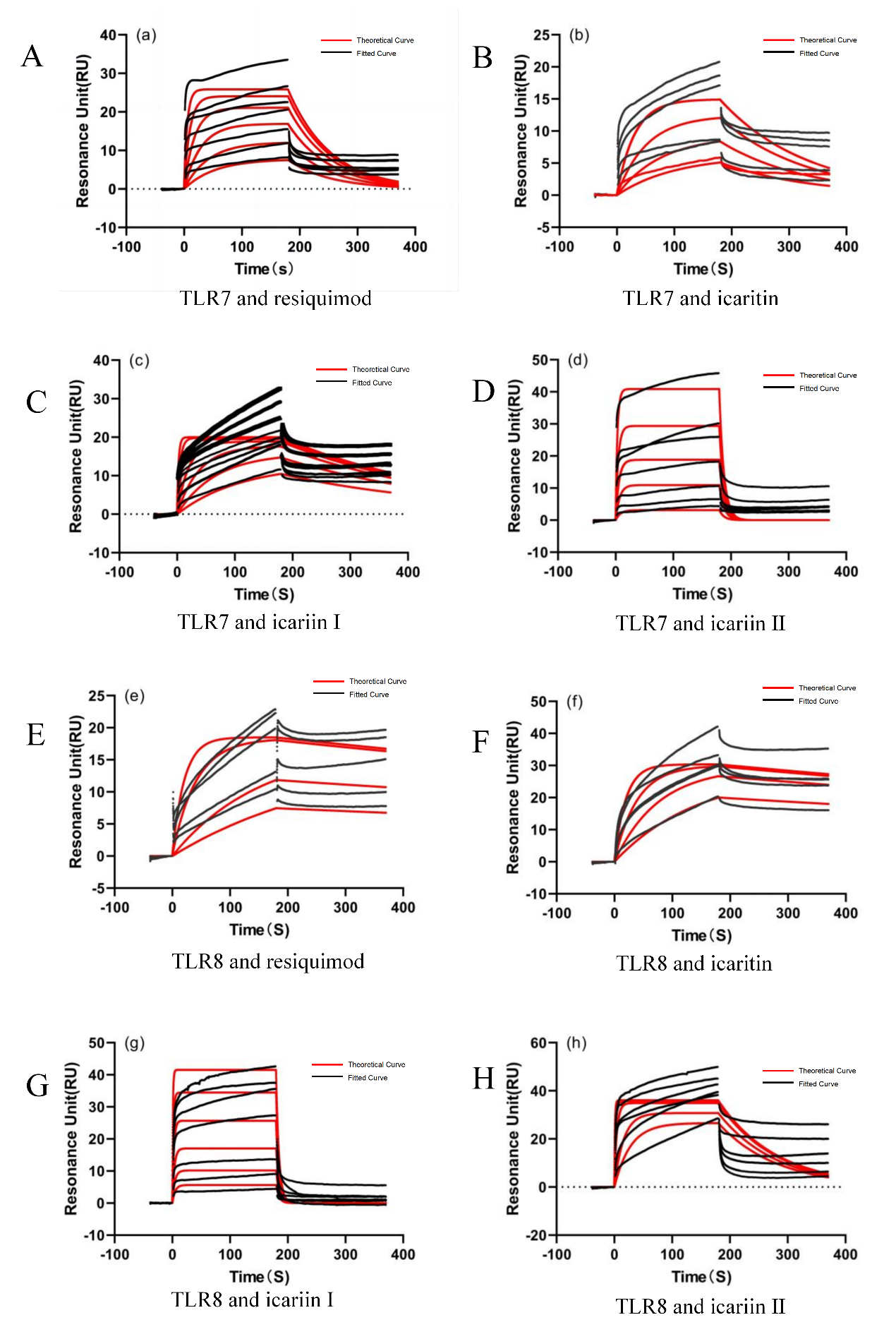


**Figure S13** Surface Plasmon Resonance (SPR) Analysis of TLR7 and TLR8 Interactions with Resiquimod, Icaritin, Icariin I, and Icariin II. (A-D) SPR response values for the interactions between TLR7 and resiquimod, icaritin, icariin I, and icariin II, respectively. (E-H) SPR response values for the interactions between TLR8 and resiquimod, icaritin, icariin I, and icariin II, respectively. In all panels, the red line represents the theoretical curves, while the black line represents the fitted curves.
